# Fine-Tuning DiffDock‑L for Allosteric Kinase Docking

**DOI:** 10.1021/acs.jcim.5c02846

**Published:** 2026-03-04

**Authors:** Eric Chen, Justin Green, Yingkai Zhang

**Affiliations:** † 5894 Simons Center for Computational Physical Chemistry at New York University, New York, New York 10003, United States; ‡ Department of Chemistry, New York University, New York, New York 10003, United States; § Department of Biology, New York University, New York, New York 10003, United States

## Abstract

Allosteric kinase inhibitors are an important modality for overcoming resistance and achieving selectivity, yet most structure-based docking and deep generative models are trained predominantly on orthosteric protein–ligand complexes. As a result, current methods often misplace allosteric kinase ligands into the adenosine triphosphate (ATP)-binding site and fail to recover the correct binding mode. Here we curate AlloSet, a kinome-wide, time-split data set of kinase–ligand complexes annotated by binding mode, to systematically evaluate and fine-tune the diffusion-based docking model DiffDock-L for allosteric pose prediction. We explore several fine-tuning strategies, including increased dropout, freezing of torsion parameters with translation/rotation-only fine-tuning, and molecular dynamics-based supersampling of receptor conformations and ligand poses. The resulting DiffDock-L-Allo model is found to markedly improve pose-recovery metrics for Type III/IV allosteric binders while preserving the performance on ATP-site ligands. Binding-mode-resolved evaluations and comparisons with cofolding models such as AlphaFold3 and Boltz-2 highlight how targeted retraining reshapes the generative model’s sampling distribution, offering practical guidance for adapting AI-driven docking to challenging, low-data binding modes in kinase structure-based drug design.

## Graphical abstract

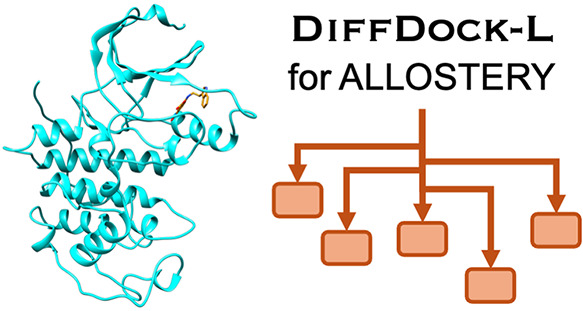

## Introduction

Kinases are an important class of proteins for their multifaceted roles in cellular function, from cell division, metabolism, immune response, signal transduction, etc.^1^ The human protein kinase family consists of 518 members with distinct functional roles, and there is a wealth of biochemical and structural data available that we can leverage.^2^ Kinases function in balance with phosphatases to regulate the signaling pathways. Kinases catalyze the transfer of a phosphate group from adenosine triphosphate (ATP) to substrates, whereas phosphatases remove these phosphate groups, thereby modulating the activity of downstream proteins. The dysregulation of kinases has long been associated with many diseases and cancers, and thus are the target of many drug discovery research campaigns, particularly for antineoplastics.^3,4^

Orthosteric inhibitors, which compete with and sterically block ATP hydrolysis at the catalytic site, are the most common FDA-approved drugs designed to target kinases. There are fewer approved allosteric inhibitors, which inhibit the kinase activity by binding to other sites such as the Type III and IV mechanisms (Figure 1).^5,6^ There has been increasing interest in recent years in the discovery of allosteric inhibitors because kinase catalytic sites tend to be highly evolutionarily conserved, which makes the risk of off-target effects and toxicity substantial for orthosteric inhibitors.^7^ Additionally, since cancer can often evolve drug resistance through point mutations, it is desirable to develop therapeutics with diverse binding modalities.^4^

**Figure 1:**
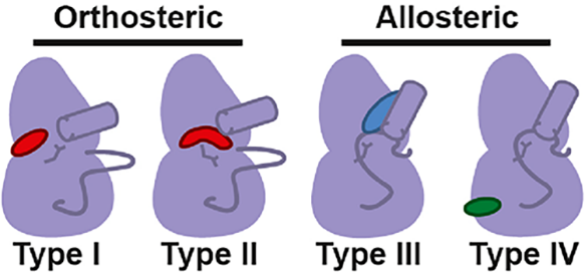
Schematic of ligand binding types. Orthosteric binders (Types I and II) bind the ATP site and are shown in red. Allosteric binders (Types III and IV) bind non-ATP sites and are colored green and blue, respectively.

Despite the advantages of allosteric modulation, many allosteric modulators have historically been discovered serendipitously, and significant challenges remain in their rational design.^7,8^ For example, the lead compounds for two clinically approved allosteric kinase drugs were identified through phenotypic assays prior to structural characterization of their binding mechanisms.^5^ Trametinib (MEK1/2; Type III) emerged from a branched-DNA assay that measured downstream protein expression,^9^ whereas asciminib (BCR-ABL; Type IV) was discovered via a differential cytotoxicity screen.^10^ Meanwhile, researchers have explored structure-based approaches to rationally target allosteric sites. For instance, Rastelli et al. and, recently, Faber et al. performed targeted virtual screens against the Type III binding site of CDK2 and confirmed the binding mode using noncompetition assays and crystal structures.^11–13^ Notably, many structure-based docking studies of allosteric ligands rely on rigid, ligand-enhanced receptor structures in which the allosteric binding pocket is already exposed. In practice, however, allosteric drug design pipelines can fail at earlier stages due to insufficient validation of the relevant pocket or because the pocket is cryptic and not apparent in available *apo* structures. In such cases, approaches that promote conformational diversity (e.g., enhanced sampling or cryptic-pocket detection methods) may be required to identify receptor conformations suitable for allosteric docking.^14–16^ These challenges stem from the complexity of modeling the diverse processes involved in protein–allosteric modulator interactions and the difficulty of developing high-throughput assays to search for allosteric binders.^17,18^

Recent trends in computational chemistry research have seen the development of deep learning-based “blind docking” methods for predicting ligand binding poses.^19–21^ Blind docking methods search for binding pockets in an unrestricted manner. This is in contrast to traditional docking methods that restrict the search space to a particular site.^22^ Blind docking algorithms may prove useful for allosteric drug development campaigns since they do not require prior knowledge of the docking site, and allow for postprediction filtering to remove orthosteric binders.^23^ To our knowledge, there has not been a systematic study comparing hit rates for allosteric ligands obtained from blind docking versus restricted-search docking focused on a predefined allosteric pocket. In this work, we focus on binders targeting the kinome because of the clinical relevance of the superfamily and the vast amount of structural data to validate our methods.

Previous work has developed a data-driven platform to benchmark the performance of docking methods within the context of its receptor conformation and ligand binding mode and highlights the challenges that current deep learning models face.^23^ We have observed that deep learning-based docking methods predominantly predict ligand binding at the orthosteric site and struggle to sample allosteric sites effectively. An empirical perspective of generative models is that they sample from a learned distribution that approximates the true data distribution. Properly designed benchmarks that split the data into distinct training and test sets to assess generalization.^24–27^ In our case, the training data contains fewer allosteric ligand–kinase complexes than orthosteric ones, and as a result, we anticipate models trained on this data to predominantly sample from these high-density regions to predict ligands bound to the orthosteric site. To make generative models useful for predicting allosteric ligands, we must extract the signal from this lower-populated data regime. To this aim, we curate a data set of kinase structures and evaluate a few fine-tuning strategies to improve sampling diversity.

It has become popular in the Large Language Model (LLM) community to take a pretrained model and fine-tune it with a relatively short training cycle on a particular task.^28^ This circumvents the extensive computational resources required to train large deep learning models from scratch and allows models to be tailored to particular tasks. We hypothesize that by fine-tuning DiffDock-L on a data set highly enriched for the types of complexes we are interested in, we can improve our performance metrics. We envision that this strategy can be particularly useful in settings where new data is iteratively introduced such as the Design-Make-Test-Analyze cycle in drug discovery.

## Methods

### Curation of AlloSet

We curate a kinome-wide structural data set of kinase binders by taking the union of the Kinase–Ligand Interaction Fingerprints and Structures (KLIFS) database^29,30^ and Modi and Dunbrack database^31,32^ (obtained on 11/20/24) (Figure S1). Notably, structures bearing allosteric ligands are restricted to a small subset of kinases, whereas orthosteric ligands predominate across the majority of the kinase targets. We rely on KLIFS for their definition of KLIFS residues, a subset of 84 structurally conserved and functionally important residues around the catalytic region selected for consistency across the kinome. We rely on the Modi and Dunbrack databases for their heuristic and structure-based definitions of the ligand binding mode. We briefly describe the heuristics below based on Aurora A Kinase residue numbering.^31,32^

- ATP binding region and hinge residues: 211–213.
- Back pocket—α C-helix and partial regions of β4–5 strands, X-DFG and DFG-Asp backbone, and DFG-Phe side chain—residues: 166–193, 196–204, 205–207, and 273–275.
- Type 2-only pocket, residues on exposed only in DFG-out structures: 184, 188, 247, and 254.

The ligand binding modes are then defined with reference to these residues. We provide additional pharmacological context to these structural heuristics as well.

- Type IV: Any small molecule in the asymmetric unit whose minimum distances from the hinge region and α C-helix-Glu­(+4) residues are both >6.5 Å.
- Type I 1/2 front: at least three or more contacts in the back pocket and at least one contact with the N-terminal region of the α C-helix.
- Type I 1/2 back: at least three or more contacts in the back pocket, but no contact with the N-terminal region of the α C-helix.
- Type II: three or more contacts in the back pocket and at least one contact in the Type II-only pocket. This binding mode traps the kinase in the inactive DFG-out conformation.
- Type III: minimum distance from the hinge >6 Å and at least three contacts in the back pocket. This binding mode traps the kinase in the α C-out conformation.
- Type I: all the ligands that do not satisfy the above criteria. This binding mode constrains the kinase in the active, α C-in/DFG-in conformation.

Type I, I 1/2, and II binders are ATP-competitive, while Type III and IV binders are noncompetitive.

We parse the binders and corresponding monomer chains from the structure. We exclude ions, crystallizing artifacts, and small molecules like glycerol by excluding compounds in the list defined in AlphaFold3 along with some manual curation.^33^ We do not remove noncanonical amino acids from the structures, but do not anticipate these noncanonical amino acids to impact our experiments because almost all ligands are >5 Å away from any noncanonical amino acid (Figure S2). The residues are renumbered according to the UniProt numbering from SIFTS.^34^ The data set is split using a 1/1/2019 time-split to follow the convention in Corso et al. where a 2019 time-split was used to train DiffDock-L (Figure 2, Table 1).^26^ It should be noted that very few kinase structures consistent with the time-split appeared in the original DiffDock-L test set (four Type I and one Type $I\frac{1}{2}$ complexes). More details about the training and splitting strategy can be found in Training and Inference. Our data set curation process is able to obtain additional complexes for the training and testing sets beyond the PDBBind data set, although the data set is imbalanced in favor of Type I binders.

**Table 1:** Breakdown of the AlloSet by Binding Mode and Time-Split

		type^a^					
	time-split date	I	$I\frac{1}{2}F$	$I\frac{1}{2}B$	II	III	IV
AlloSet	<1/1/2019	3082	62	203	257	90	119
	≥1/1/2019	903	37	80	97	49	89
total		3985	99	283	354	139	208

a F: Front; B: back.

**Figure 2:**
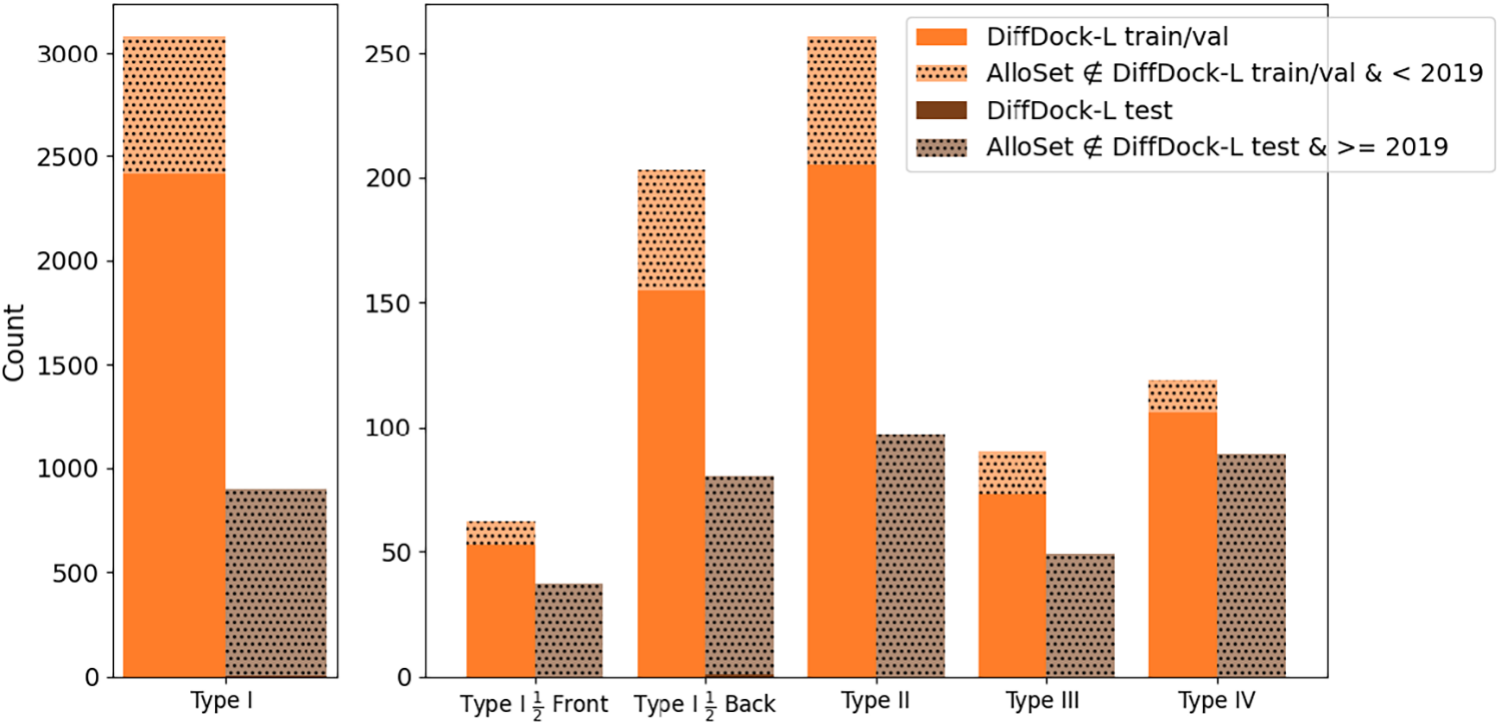
Time split and distribution of AlloSet binding modes as defined by the Modi and Dunbrack heuristics. The AlloSet is time-split on 1/1/2019, and the dotted bars indicate the number of new poses added during the curation process.

For the fine-tuning data set, we show the distribution of kinase–allosteric ligand type pairs and note that pairs present in the test set are typically also represented in the training set (Figure 3). We additionally report the distribution of select molecular properties of the Type III and IV ligands and observe that the chemical properties of the test split are well represented by the training split (Figure 4). To define and compare allosteric pockets in a site-centric manner (rather than relying solely on conformation-based “Type” labels), we leveraged the pocket annotations from the Laufkötter data set, which manually assigns structure-informed pocket labels to kinase–ligand complexes.^16,35^ AlloSet is a superset of the Laufkótter data set, and for structures present in both data sets, we report the distribution of assigned pocket labels. We observe that our training/validation set spans a diverse collection of deep pockets (B–H), well-characterized shallow pockets (C–F), and more sparsely characterized shallow pockets (G–L) (Table S1). Importantly, the pocket labels represented in the test split are also well represented in the training split, indicating that evaluation is not driven by unseen pocket categories.

**Figure 3:**
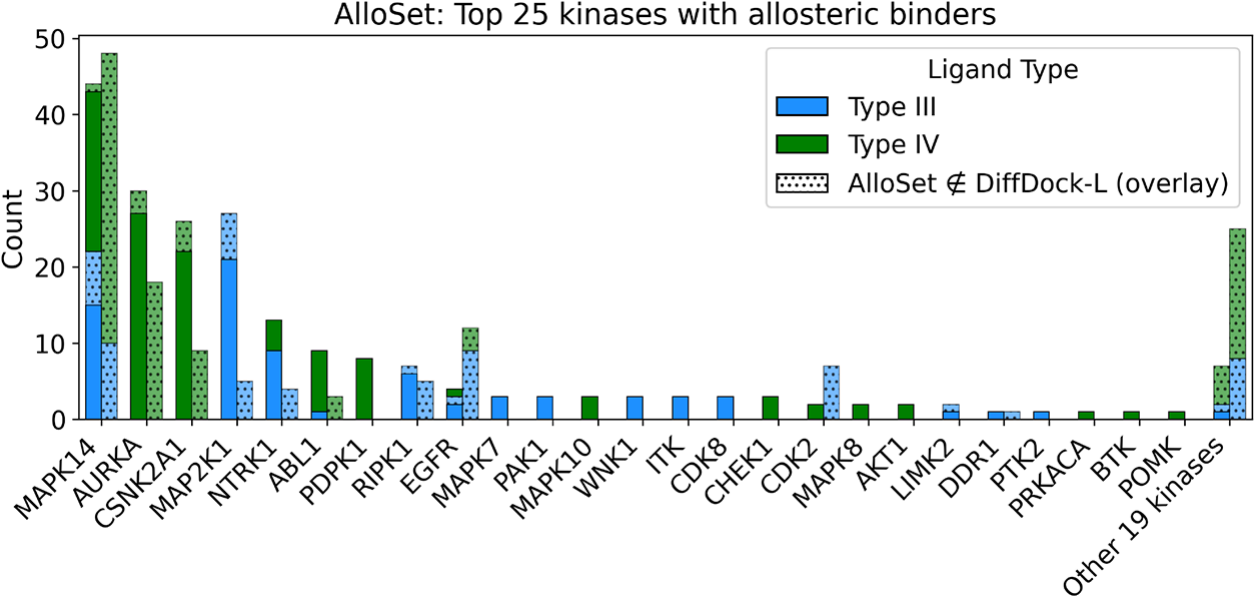
Top 25 kinases bound by Type III or IV binders split by prevalence in our training and validation (left bar) or test (right bar) sets. The dotted bars indicate new data points obtained during our curation process.

**Figure 4:**
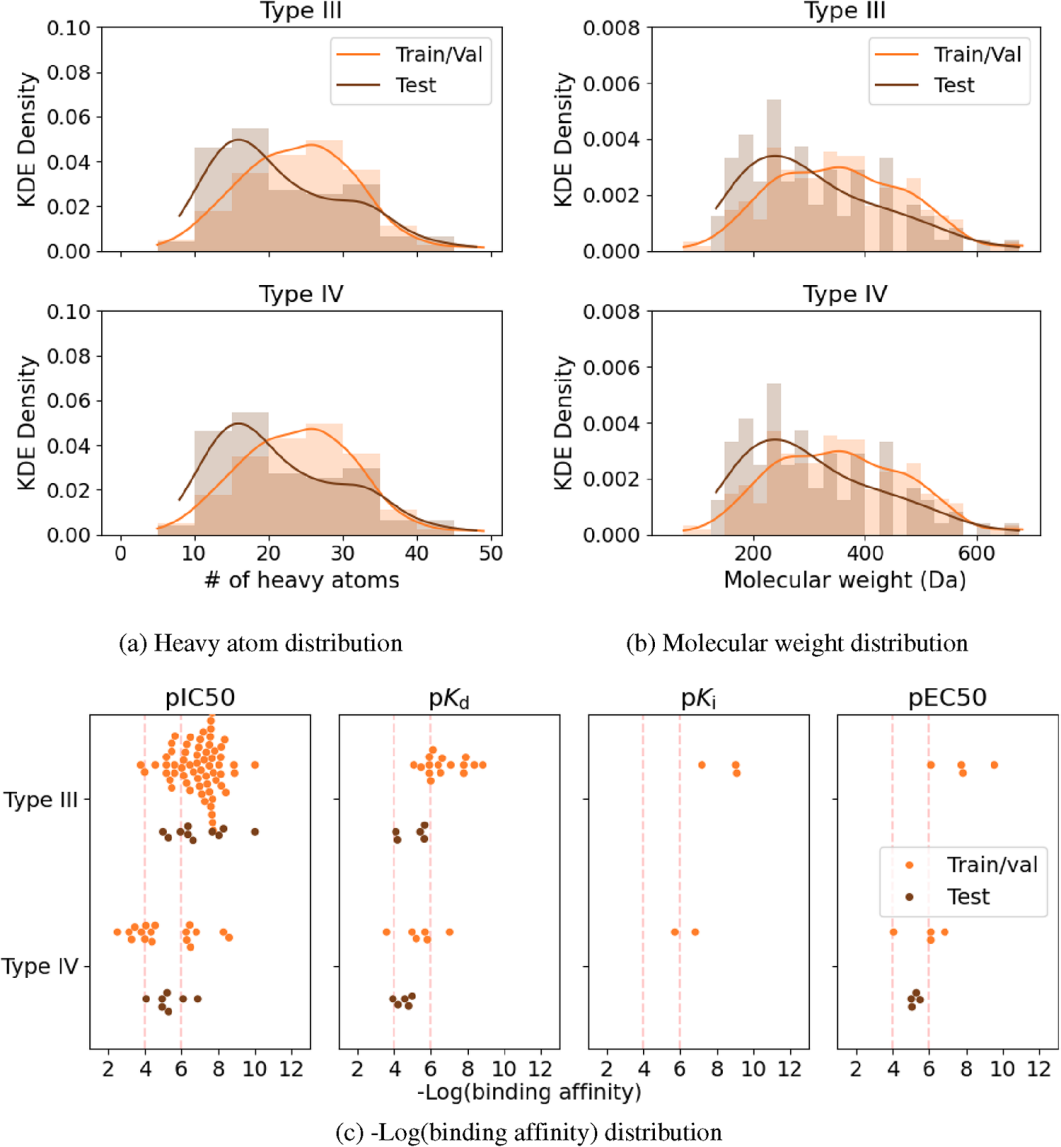
(a) Heavy atom, (b) molecular weight, and (c) log-transformed binding affinity (−log­(BA)) distribution of the Type III and IV ligands in the training and test splits. A kernel density estimation determines the indicated probability density distribution of the heavy atom and molecular weights. The dotted red lines at −log­(BA) = 4 and =6 reflect the thresholds for weak and medium/strong binders, respectively.

#### Ligand Diversity

To quantify the diversity of our allosteric ligand data set, we first cluster our ligands by structural similarity and then quantify the per-cluster α diversity. For clustering, we used RDKit^36^ to generate 2048-bit, radius 3 Morgan fingerprints^37^ and clustered these fingerprints using the Butina algorithm.^38^ We use the Tanimoto distance metric and similarity threshold value of 0.8.^38^ This procedure clusters our 346 ligands into 287 clusters and highlights that the vast majority of ligands are placed in singleton clusters, and a small number of other clusters contain less than 10 ligands (Figure 5).

**Figure 5:**
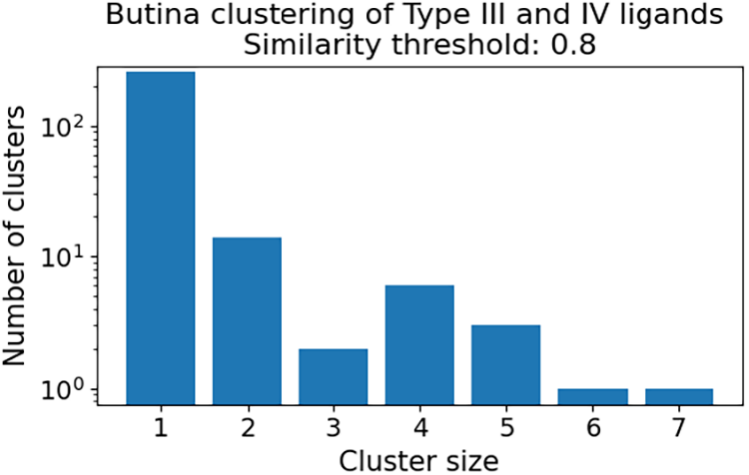
Logarithmic histogram showing cluster sizes created by Butina clustering.

We also compute the Shannon Index of our clusters to quantify the “richness” of our data set (Table 2).^39^ The Shannon index is given by the formula [Disp-formula eq1]

1 $$H=-\sum_{i=1}^{S}p_{i}\;\log_{2}\left(p_{i}\right)$$

where *S* is the total number of clusters and *p*_*i*_ is the relative proportion of ligands belonging to cluster *i*.^39^ Note that the maximum diversity value for the Shannon index of this data set (i.e., every ligand has a Tanimoto similarity of less than 0.8 with every other ligand) would be log_2_(346), or 8.43 bits. From this table, we can see that our test and training folds all exhibit similar high levels of diversity, supporting the notion that model evaluation is not driven by a narrow scaffold family present disproportionately in one split.

**Table 2:** Breakdown of Shannon Indices for Butina Clustering

split	Shannon index (bits)
Type III train/val	6.30
Type IV train/val	6.94
Type III test	5.09
Type IV test	5.89

#### Hydrogen Correction

We initially observed inconsistencies with how ligand features, such as bond type, aromaticity, and formal charge, are determined and attributed it to the ambiguities in the ligand preprocessing stage. To address this, we perform a preprocessing step that explicitly defines hydrogen positions in the ligand files. For each ligand in the AlloSet, we use OpenBabel to predict hydrogens at a pH of 7.4. Then, we use RDKit to check for the proper hydrogenation and charge definitions of imidazole, amino, nitro, tetrazole, and carboxyl groups and correct them if they contain chemical violations.^36,40^ Finally, a “corrected” ligand file is produced with explicit hydrogens.

### DiffDock

In its initial release, DiffDock achieves a state-of-the-art performance of 38% root mean square deviation (RMSD) < 2 Å success rate on the PDBBind test set, but its more recent and larger adaptation, DiffDock-L, surpasses it with a 43% success rate.^20,26^ The major advancement in DiffDock is its treatment of molecular docking as a diffusion generative modeling problem. A diffusion model takes a noisy prior distribution as input and uses a neural network, called the “score model”, to iteratively “denoise” the prior distribution into samples of some target distribution. In this case, the noisy prior distribution is a ligand–protein complex where the ligand is located randomly in space, and the samples generated will place the ligand in a binding pocket. Rather than diffusing in the product space of the three-dimensional (3D) coordinates of every atom in the ligand, DiffDock holds bond lengths, angles, and rings rigid, and the diffusion process is defined over the “ligand pose manifold” $M_{c}$ (space of all possible ligand poses). The authors postulate that this manifold corresponds directly to the simpler product space P of the translational (tr; $T^{3}$ ), rotational (rot; SO(3)), and torsional (tor; SO(2)^*m*^) degrees of freedom of a ligand, where *m* is the number of possible torsion angles (eq 2).

2 $$A_{c}^{-1}:M_{c}\to P=T^{3}\times \mathrm{SO}\left(3\right)\times \mathrm{SO}{\left(2\right)}^{m}$$

#### Diffusion Models

The core model principle is to train a denoising score-matching model, which represents a manifold with a score function (or the gradient of the log probability density function) (eq 3).^41^

3 $$s_{\theta }\left(x\right)\approx \nabla_{x}\;\log p\left(x\right))$$

During the training process, samples *x* from the training data set are made noisy using the traditional forward diffusion process with a stochastic differential eq 4

4 dx=f(x,t)dt+g(t)dw,⁣t∈(0,T)

where *w* is the Wiener process or Brownian motion, *f* is the drift function, and *g*(*t*) is the diffusion coefficient. When we approach a large *T*, the ending distribution *p*_*T*_(*m*) approaches a simple Gaussian noise. This ending noisy distribution is then the prior that we sample from during reverse diffusion. The reverse diffusion process is formalized by the eq 5

5 $$dx=\left[f\left(x_{t},t\right)dt-g^{2}\left(t\right)\nabla_{x}\;\log p_{t}\left(x\right)\right]dt+g\left(t\right)d\mathrm{w̅}$$

where ∇_*x*_ log *p*_*t*_(*x*) is the “score function” of our target probability distribution, and is estimated by the neural network. Thus, during inference of the ligand pose, we can sample our target probability distribution using a geodesic random walk with the score as the drift term.^42^

In practice, reverse diffusion during inference amounts to obtaining the relative translation, rotations, and torsion angles updates from a learned distribution that was trained on how to denoise (reverse diffuse) these updates.

#### Model Architecture

DiffDock uses a message-passing heterogeneous graph architecture that relies on SE(3)-equivariant convolutional networks as implemented in E3NN.^43^ The score model embeds proteins with Cα as nodes and ESM-2 language model embeddings,^44^ and embeds ligand heavy atoms as nodes and physicochemical features. The edges are defined based on distance cutoffs that depend on node type and diffusion time. The DiffDock-S and -L models improve the score model architecture compared to DiffDock for greater depth without increasing the runtime, and simplify and balance the confidence model architecture and training.^26^

The E3NN framework is built into the interaction and output layers of the model through tensor product convolutions to ensure equivariance for data/training efficiency and generalization.^43^ In other words, E3NN ensures that the samples transform in a predictable way even when the inputs undergo rotational/translational/reflection (SE(3) group) changes.

#### Confidence Model

Finally, a trained confidence model uses an all-atom representation based on standard PDB atom-typing conventions as input to rank the best binding pose. To train the confidence model, the trained score model generates poses for every training example and labels each pose if the RMSD < 2 Å. The confidence model is then trained with cross-entropy loss to predict a scalar representing the binary classification of the pose being correct or incorrect.

### Training and Inference

While Corso et al. draw both their training and validation set from their pre-2019 time split for training DiffDock-L, we draw our fine-tuning training set from the pre-2019 time split and the validation set from the post-2019 time split to avoid contaminating our validation set with samples that are used in training DiffDock-L (Figure 2, Table 3).^26^ For testing, we deduplicate examples with the same UniProt ID and ligand name, and then use the 844 Type I and a stratified random selection of 34 Type III and 62 Type IV crystal structures after the 1/1/2019 time split as our test set. The remaining 15 Type III and 26 Type IV structures after the time split and not used for testing are used as the validation set.

**Table 3:** Table of the Train, Validation, and Test Set Size used in our Experiments, Compared to the Boltz-2 and AlphaFold3 Test Set

		type^a^					
	time-split date	I	$I\frac{1}{2}F$	$I\frac{1}{2}B$	II	III	IV
fine-tune train	<1/1/2019					90	119
fine-tune val	≥1/1/2019					15	26
fine-tune test	≥1/1/2019	844				34	62
Boltz-2/AlphaFold3 test	>9/30/2021	309				8	32

a F: Front; B: back.

For validation and testing, we generate 10 complexes for a given receptor–ligand pair, and then use the confidence model to rank them. We track our performance using two main error metrics: the symmetry corrected RMSD between ligand atoms and their ground truth positions (“RMSD”), and the Euclidean distance between the center of the ligand and its ground truth center (“Centroid”).^45^ We track these metrics both for the top-ranked complex (“Top 1”), and the best value for any of the 10 complexes generated (“Any”). We define a prediction as successful in a given metric when the value is less than 2 Å. For all results, we report a 95% confidence interval using the Wilson score interval and indicate statistically significant differences with the baseline where the *p* value <0.05.^46,47^

### Sampling Temperature

DiffDock-L has an optional “temperature sampling” procedure that is configured by default during inference. When computing the perturbations of the ligand at each time step (i.e., Δ*r*, Δ*R*, and Δθ), the procedure is modified for temperature sampling from the original algorithm to the following (eq 6):

6 $$\epsilon_{\mathrm{tr}}\leftarrow e^{\epsilon_{\mathrm{tr}0}\ln\left(\sigma_{\mathrm{maxtr}}\right)+\left(1-\epsilon_{\mathrm{tr}0}\right)\ln\left(\sigma_{\mathrm{mintr}}\right)}$$

7 $$\lambda_{\mathrm{tr}}\leftarrow \frac{\epsilon_{\mathrm{tr}}+\sigma_{\mathrm{tr}}}{\epsilon_{\mathrm{tr}}+\frac{\sigma_{\mathrm{tr}}}{T_{\mathrm{tr}}}}$$

8 $$\Delta r\leftarrow \Delta \sigma_{\mathrm{tr}}^{2}\left(\lambda_{\mathrm{tr}}+T_{0}\frac{\psi_{\mathrm{tr}}}{2}\right)\alpha +\sqrt{t\left(1+\psi_{\mathrm{tr}}\right)}z_{\mathrm{tr}}$$

9 $$\epsilon_{\mathrm{rot}}\leftarrow e^{\epsilon_{\mathrm{rot}0}\ln\left(\sigma_{\mathrm{maxrot}}\right)+\left(1-\epsilon_{\mathrm{rot}0}\right)\ln\left(\sigma_{\mathrm{minrot}}\right)}$$

10 $$\lambda_{\mathrm{rot}}\leftarrow \frac{\epsilon_{\mathrm{rot}}+\sigma_{\mathrm{rot}}}{\epsilon_{\mathrm{rot}}+\frac{\sigma_{\mathrm{rot}}}{T_{\mathrm{rot}}}}$$

11 $$\Delta R\leftarrow \Delta \sigma_{\mathrm{rot}}^{2}\left(\lambda_{\mathrm{rot}}+T_{0}\frac{\psi_{\mathrm{rot}}}{2}\right)\beta +\sqrt{t\left(1+\psi_{\mathrm{rot}}\right)}z_{\mathrm{rot}}$$

12 $$\epsilon_{\mathrm{tor}}\leftarrow e^{\epsilon_{\mathrm{tor}0}\ln\left(\sigma_{\mathrm{maxtor}}\right)+\left(1-\epsilon_{\mathrm{tor}0}\right)\ln\left(\sigma_{\mathrm{mintor}}\right)}$$

13 $$\lambda_{\mathrm{tor}}\leftarrow \frac{\epsilon_{\mathrm{tor}}+\sigma_{\mathrm{tor}}}{\epsilon_{\mathrm{tor}}+\frac{\sigma_{\mathrm{tor}}}{T_{\mathrm{tor}}}}$$

14 $$\Delta \theta \leftarrow \Delta \sigma_{\mathrm{tor}}^{2}\left(\lambda_{\mathrm{tor}}+T_{0}\frac{\psi_{\mathrm{tor}}}{2}\right)\gamma +\sqrt{t\left(1+\psi_{\mathrm{tor}}\right)}z_{\mathrm{tor}}$$

where the ϵ, *T*, and ψ variables are new configurable values.^26^ We note that DiffDock disables temperature sampling for validation and enables temperature sampling for inference runs. We question whether temperature sampling might be ill-suited for our data set and experiment with simply disabling it altogether.

### Fine Tuning

From the data set we collated, we chose the 209 Type III and IV complexes that have been uploaded to the PDB before 2019 to use as our training set and 41 of the post-2019 Type III and IV complexes to use as a validation set to track training progress (Table 3). Using the pretrained DiffDock-L model distributed by Corso et al., we fine-tune the model with this new data set for 5000 epochs using a learning rate of 0.0001.^26^ We monitor our RMSD metrics of the fine-tuned model on our validation set every 100 epochs and save the model parameters when the rolling average “Any RMSD” metric of the last 10 validation runs (1000 epochs) is at their best. Progress is monitored using the “Weights and Biases” platform.^48^ The various experiments are described in detail below and are summarized in Table 4.

**Table 4:** Comparison of the Hyperparameters of the Fine-Tuning Experiments

run name	α	β	torsion loss	dropout	data set size	Temp sampling
baseline	N/A	N/A	N/A	N/A	N/A	true
baseline no Temp	N/A	N/A	N/A	N/A	N/A	**false**
fine tuning	1.0	1.0	0.33	**0.1**	209	true
dropout	1.0	1.0	0.33	0.3/**0.5**/0.7	209	true
MD supersample	1.0	1.0	0.33	0.5	**627**	true
tr/rot only	**1.25**	**2.25**	**0.0**	0.5	209	true

### Dropout

Compared to the 17k complex PDBBind data set used to pretrain DiffDock-L, our fine-tuning training data set is more than an order of magnitude smaller.^26,49^ Because of this discrepancy, we have concerns that our fine-tuning will lead to overfitting. One common strategy for addressing overfitting is “dropout”, which involves randomly setting some population of neurons to 0 during training.^50^ By default, DiffDock-L’s training script uses a 10% dropout rate, so we experiment with increasing the dropout rate to 30, 50, and 70%.

### Molecular Dynamics Supersampling

Similarly to Boltz-2, we also try to combat overfitting by augmenting our data set with snapshots of MD simulations of our experimentally determined structures.^51^ For this purpose, we use AMBER 2023 to run 300 K, 2 ns explicit solvent (TIP3P) simulations of each complex in our training set.^52^

Missing regions in receptor structures are first corrected using the procedure outlined in Supporting Methods. Ligands are hydrogenated using the procedure outlined in Hydrogen Correction. Receptors are protonated using PDB 2PQR.^53^ Ligand parametrization is done using the GAFF2 force field.^54^ Input file preparation was handled by the Antechamber toolkit.^55^ Simulations are performed using the FF14SB force field.^56^ Energy minimization is first performed with restraints added to receptor and ligand atoms of 2 kcal/mol. Heating is performed from 10 to 300 K over 50 ps with 2 kcal/mol restraints again added to receptor and ligand atoms. Density equilibration is done at 300 K for 50 ps with a pressure coupling constant (taup) of 1.0, and the same restraints as the energy minimization and heating steps. Two 100 ps equilibration steps are performed before the final MD run: the first with the receptor and ligand restraints reduced to 0.5 kcal/mol and the second without restraints.

For each simulation, we select two frames at random that have a ligand RMSD < 2 Å compared to the ground truth crystal structure, effectively tripling the size of our training data set. By only selecting frames with a ligand RMSD of less than 2 Å, we exclude dissociated complexes and restrict the augmented set to conformations that preserve the binding mode of the reference structure, thereby maintaining protein–ligand interaction (PLI) patterns consistent with the original complex. It should be noted that the purpose of these MD-derived frames is not to discover rare binding modes, but rather to provide physically plausible local fluctuations of the protein–ligand complex around the experimentally observed pose (e.g., side-chain and backbone adjustments within the binding site). Our objective from this kind of augmentation was to regularize fine-tuning and improve robustness by exposing the model to a small ensemble of near-native conformations rather than a single static structure.

### Translation and Rotation Loss Only

DiffDock-L samples *t* from a uniform distribution when computing the score-matching loss function for training and then sums together three subloss functions: one for translation, one for rotation, and one for torsion scores. DiffDock-L provides weighting parameters for the final loss computation, but by default sets all three weights to 0.33.^20,26^ We try a different strategy called tr/rot_only, where we train a model to sample *t* from a β-distribution function with α = 1.25 and β = 2.25 (eq 15) to bias the early stages of reverse diffusion and set the torsion loss to 0.0.

15 $$f\left(x;\alpha ,\beta \right)=\frac{x^{\alpha -1}{\left(1-x\right)}^{\beta -1}}{B\left(\alpha ,\beta \right)}$$

where

16 $$B\left(\alpha ,\beta \right)=\int_{0}^{1}t^{\alpha -1}{\left(1-t\right)}^{\beta -1}\;dt$$

### Cofolding

For both AlphaFold3 and Boltz-2, we generate and rank 5 samples for each complex using default generative and confidence model parameters. We use the Open-structure tools to calculate the protein– and protein–ligand interaction (PLI) local distance different test (lDDT) scores.^57,58^ To calculate the RMSD and centroid distances for the cofolding results, we first perform a global sequence alignment with Biopython to obtain consistent residue numbering and then perform an all-Cα superimposition.^59,60^ It should be noted that the test set size for the cofolding method is much smaller than the DiffDock-L because AlphaFold3 and Boltz-2 use a 9/30/2021 time-split while DiffDock-L use a 1/1/2019 time-split (Table 3).

## Results and Discussion

### Fine-tuning DiffDock-L Improves Docking Predictions

As a baseline, we evaluate the performance of DiffDock-L with temperature sampling on the AlloSet test set when predicting 10 samples per complex using only the Type III and Type IV ligands in our curated data set before 1/1/19, consistent with the DiffDock-L training set (Figure 6). We use two metrics, RMSD and centroid distance between the predicted pose and the reference crystal structure ligand.^45^ We denote the fraction of complexes that sampled any pose that satisfy the metric with the Any label, and the best confidence-model-ranked performance with the Top 1 label. Notably, we observe a significant drop in performance when docking Type III and IV ligands compared with the Type I ligands. The centroid distance results suggest that the vast difference in performance occurs because the docking model is unable to frequently sample the allosteric poses. These results are consistent with the imbalanced training set favoring orthosteric over allosteric binders (Figure 2) and with our observations in our previous work.^23^ A common inference strategy tunes the temperature sampling hyperparameters to adjust the quality and diversity of the predictions. When we turn off temperature sampling, we observe that it makes little difference in our success metrics, suggesting this may be an unnecessary step for some data sets.

**Figure 6:**
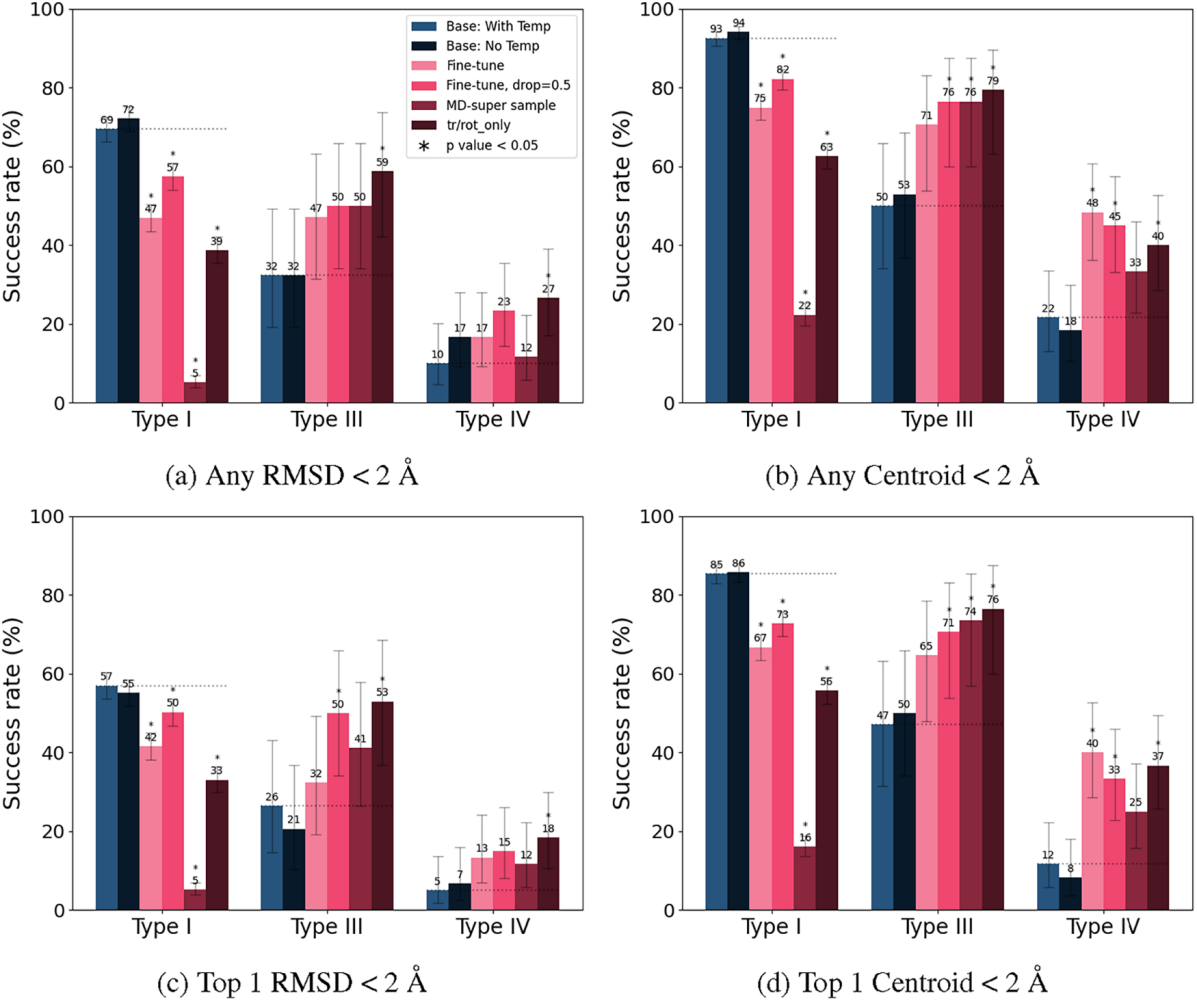
Fraction of DiffDock-L predicted complexes in the test set that satisfy the requirements (a) if any of the 10 sampled poses that result in RMSD < 2 Å or (b) centroid distance <2 Å and (c) if the top 1 confidence poses result in RMSD < 2 Å or (d) centroid distance <2 Å. The blue bars indicate the baseline DiffDock-L models with and without temperature sampling. The red bars indicate the various fine-tuning strategies such as with only the allosteric training set with dropout = 0.1 or 0.5, MD-supersampling, and updating only the translation and rotation heads and freezing the torsion head (tr/rot_only). The dotted line indicates the performance of DiffDock-L baseline, error bars denote 95% confidence intervals, an asterisk (*) denotes statistical significance (*p* < 0.05) compared to the baseline.

In all cases shown, fine-tuning is able to improve the prediction of the Type III and IV binding pose compared to the baseline with varying magnitudes of performance decreases in the prediction of the Type I binding pose. The fine-tune dropout = 0.5 and tr/rot_only strategies roughly double the baseline performance, with tr/rot_only performing best in predicting the Type III and IV binding mode with RMSD < 2 Å space in any of the 10 samples (Figure 6a,c) and with fine-tune dropout = 0.5 performing best in predicting the Type IV binding mode with centroid <2 Å space (Figure 6b,d). Due to the limited training data, we hypothesized that we could augment our training data by supersampling our data set with snapshots of near-native conformations from MD simulations, but we do not see any advantages over fine-tuning with just the crystal structures. For Type IV binders, we observe encouraging improvements in centroid performance, indicating that the models are finding the correct site, but further improvement can be made to predict the exact pose (Figure 6b,d). The intuition behind the DiffDock-L model is that the early stages of reverse diffusion explore the binding sites on the receptor through the translation and rotation updates, and the later stages position the ligand in the binding through rotation and torsional updates. The slight improvement we see from the tr/rot_only strategy lends support to the hypothesis that focusing our fine-tuning efforts on only translation and rotation could improve the frequency with which DiffDock-L selects the correct binding pocket.

Virtual screening for allosteric compounds is a natural setting where blind docking models may improve over traditional docking methods for the potential to use postprediction filtering to remove orthosteric poses. In this setting, we want a balanced performance across both orthosteric and allosteric poses. We find that all fine-tuned models lead to a decrease in performance on Type I ligands (Figure 6). Of the methods tested, fine-tuning with dropout = 0.5 results in the lowest reduction of Type I performance. We additionally tested dropout = 0.3 and =0.7, but 0.5 remained the best overall performing model (Figure S3). Including Type I validation set performance as another early stopping criteria can counter this trade-off that we observe. Interestingly, the largest decrease in performance for Type I poses occurs when we supersample the training set with poses from MD simulations. We speculate that a large increase in the number of new training data points leads to catastrophic forgetting, and fine-tuning the data set size may be another hyperparameter to tune for balanced performance.

Once a model is developed to sample diverse binding sites, the next step is to rank and discriminate between the predicted binding modes. Even if the right binding site is sampled, a scoring function that can discriminate between the correct and false poses is needed. DiffDock-L introduces a confidence model that ranks the predicted protein–ligand binding pose. Overall, we do not observe large drops in performance when selecting the top-ranked pose, suggesting that the confidence model may be well calibrated for identifying allosteric kinase poses (Figure 6). To support this claim, further work is needed such as the development of a robust scoring function assessment with computationally generated allosteric decoys set (allosteric ligands in the orthosteric site and orthosteric ligand in allosteric sites).^61–64^ Furthermore, a target-specific classifier model can be trained on this data for greater screening performance.^65^

### Cofolding Methods Still Have Challenges

We also evaluate Boltz-2 and AlphaFold3 to jointly predict the protein–ligand binding mode on our data set (Figures 7 and 8). It should be noted that our test set size is much smaller because AlphaFold3 and Boltz-2 use a 9/30/2021 time-split, while DiffDock-L uses a 1/1/2019 time-split (Table 3). To assess each complex, we supply the protein sequence and hydrogen-corrected SMILES as a ligand, and evaluate the protein– and PLI–lDDT scores. On this set, we observe that Boltz-2 and AlphaFold3 lead to a drop off in performance similar to DiffDock-L baseline when predicting allosteric poses compared to orthosteric poses in the RMSD, centroid, and PLI–lDDT scores despite high protein–lDDT (>0.8) (Figures 7 and 8a). Based on the RMSD and centroid, the DiffDock-L baseline performs worse than the Boltz-2 on the Type I and Type IV binding modes (Figure 7c,d), but our fine-tuned models still have superior performance in predicting Type III poses (Figure 7a). We also evaluate the cofolding methods using the PLI–lDDT scores, a metric that is more amenable to differences in receptor conformations. In this metric, Boltz-2 and AlphaFold3 perform comparably across all ligand binding modes (Figure 8b).

**Figure 7:**
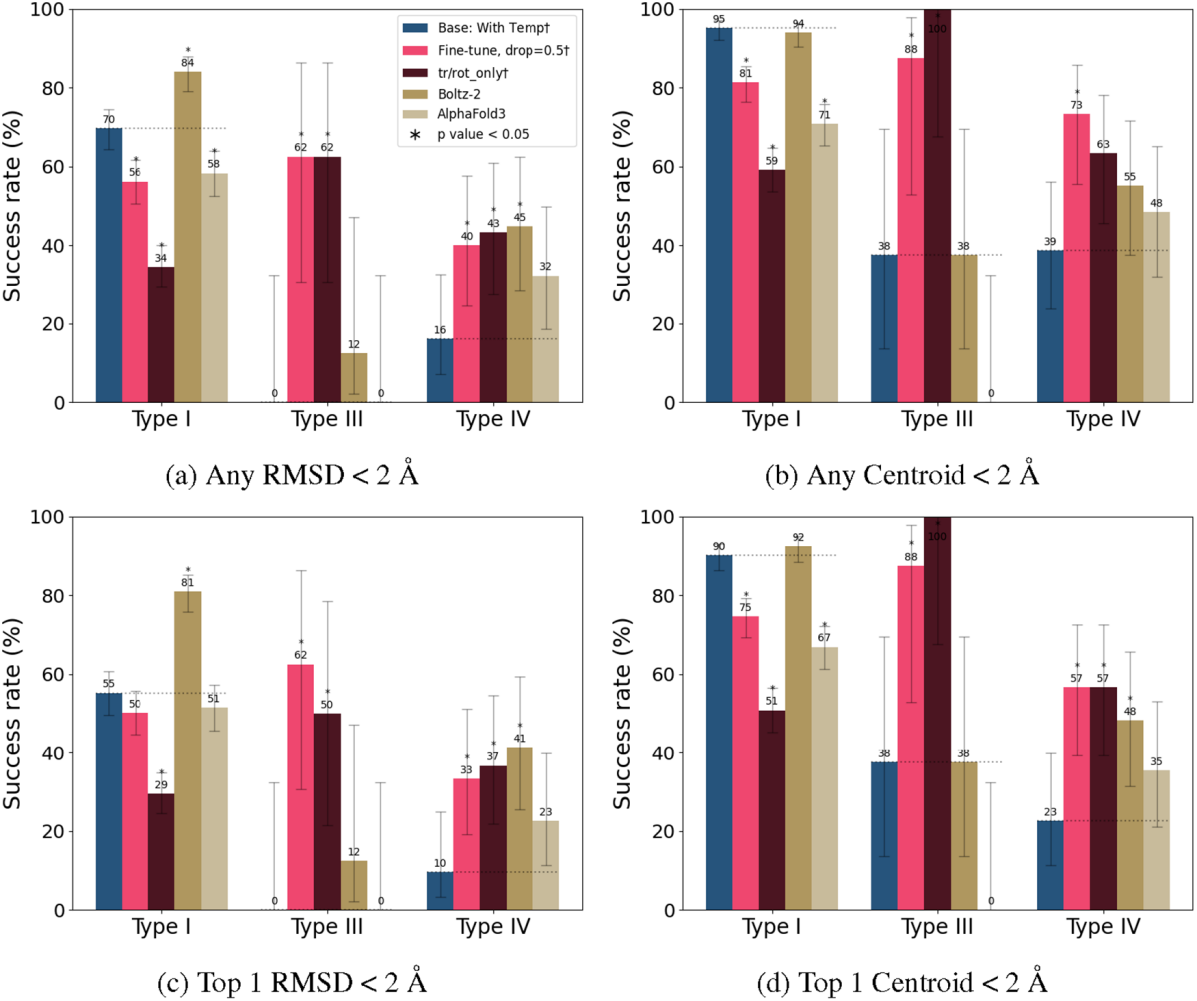
Fraction of the Boltz-2 and AlphaFold3 cofolding methods that satisfy the requirements (a) if any of the 10 sampled poses result in RMSD < 2 Å or (b) centroid distance <2 Å and (c) if the top 1 ranked poses result in RMSD < 2 Å or (c) centroid distance <2 Å. For the cofolding methods, we apply an all-Cα superimposition prior to calculating the RMSD and centroid distance. The dotted line indicates the performance of DiffDock-L baseline, error bars denote 95% confidence intervals, an asterisk (*) denotes statistical significance (*p* < 0.05) compared to the baseline condition. ^†^Note that the DiffDock-L and fine-tuned results shown here reflect the later 9/30/2021 time-split.

**Figure 8:**
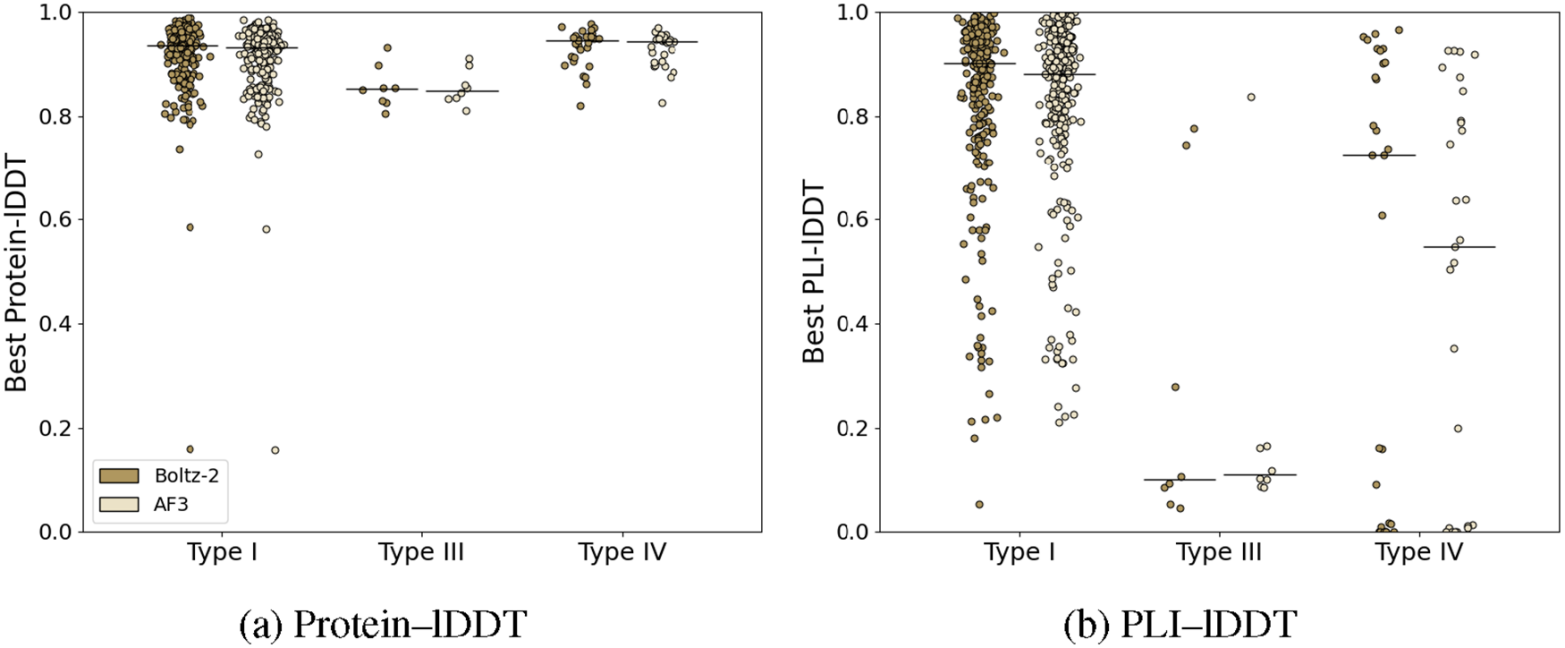
Strip plots of the best global (a) protein and (b) PLIlDDT scores of AlphaFold3 and Boltz-2 on the test set across five samples. The line indicates the median performance for a particular binding mode.

Although recent advances in cofolding methods are impressive, these results highlight that challenges remain when predicting allosteric ligands compared to orthosteric ligands. This observation with cofolding models is also consistent among broader orthosteric and allosteric data sets.^66,67^ Leveraging fine-tuning tactics on cofolding models will likely yield similar improvements in performance on tailored data sets, such as for allostery. Such strategies are being explored at scale in industry through federated learning of Boltz-2 and OpenFold3.^33,68,69^

### Fine-Tuning Improves Pocket Selection

Preliminary observations indicate that one common failure mode of DiffDock-L with respect to allosteric kinase ligands is that the model would inappropriately dock allosteric ligands into the orthosteric pocket. To see how widespread this phenomenon is, we visualize the predicted docking modalities. This is challenging because the complexes we tested have different receptor structures, and there is no straightforward way to superimpose the 3D structures of the predicted poses. To address this, we devised a fingerprint for the kinase–ligand binding location and visually represented it with a principal component analysis (PCA) plot. For each kinase in the data set, we selected 84 “KLIFS residues” that are highly conserved among kinases and took the minimum residue–ligand distance vector as our fingerprint.^23,30^

We first use this procedure to fingerprint ground truth crystal structures. We randomly select 100 orthosteric, 100 Type III, and 100 Type IV complexes as training data for PCA and find the centroids of each Modi and Dunbrack complex type in PCA vector space (Figure S4).^31,32^ We then fingerprint and PCA project the top-1 predicted Type III and IV test set results from the baseline and tr/rot_only fine-tuned models onto this crystal ligand binding location embedding space (Figure 9). Each of these predicted poses is assigned a marker corresponding to the ground truth label and a color corresponding to the ligand type label of the nearest centroid. If a docking model always docks a ligand into the correct pocket, we expect Figure 9 to only contain blue dots and hollow green squares. Any other combination of color and shape indicates that the model selected the wrong pocket as defined by our fingerprinting procedure. On one hand, Figure 9 above shows many red dots and hollow red squares, indicating that the baseline DiffDock-L model frequently docks allosteric ligands into the orthosteric pocket. On the other hand, Figure 9 below shows fewer red markers and more blue markers and hollow green squares, which indicates a qualitative improvement in the capability of the fine-tuned models to predict the correct binding pocket.

**Figure 9:**
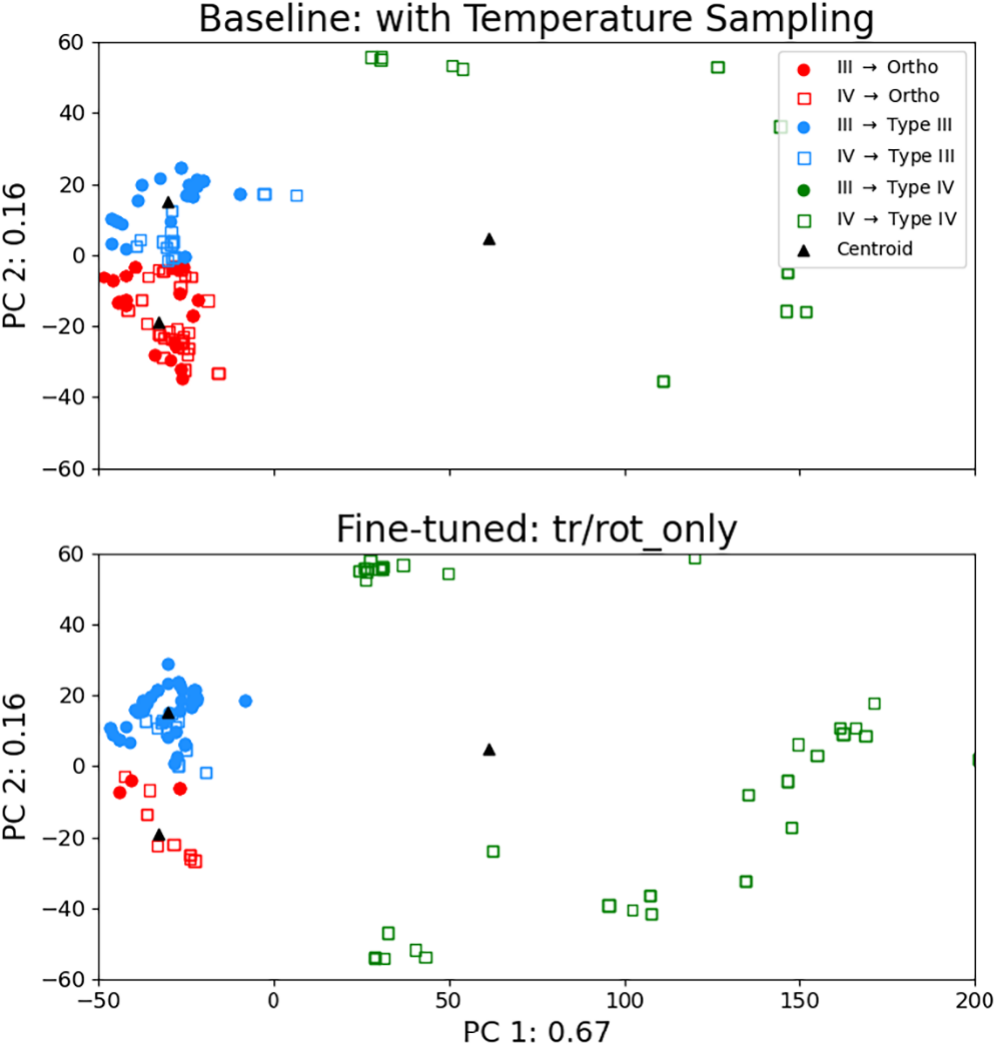
Kinase–ligand predicted pose location representation space from the baseline DiffDock-L and the tr/rot_only fine-tuned models projected onto the crystal ligand location representation space (Figure S4). The *X*- and *Y*-axis reflect the first and second principal components, respectively, and are labeled with the explained variance ratio from PCA of the crystal ligand binding locations. The shape of the marker indicates the actual binding modality of the ligand. Filled circles are Type III binders, and hollow squares are Type IV binders. The color of the mark indicates the predicted binding modality. Red markers indicate predicted orthosteric binding, while green and blue markers indicate predicted Type III and Type IV binding, respectively. The black triangles indicate the centroids of each binding modality as defined by the PCA training procedure.

## Conclusion

A key challenge in generative modeling for protein–ligand interactions is determining whether a model trained on known interactions can generalize to predict novel binding poses of distinct compounds at different sites or proteins. Commercial value from drug discovery comes from the ability to target novel proteins and expand to novel modalities or chemical space not previously patented or studied. Some researchers suggest that DiffDock-L excels only on proteins represented in the training set.^70^ Other work has discussed the idea that deep learning cofolding models are overfit to specific protein families and that the success rate is dictated by the similarity of structures to the training set.^25,71^ While the proteins in our test set are well-represented in the training set, we ask a distinct question on one particular protein superfamily: can we shift the trained distribution of a generative model to better predict binding poses at binding pockets underrepresented in the training data (Figure 3)?

Allosteric kinase inhibitors remain challenging for structure-based docking because most modern deep learning docking models are trained predominantly on ATP-site complexes and therefore tend to oversample orthosteric poses. To enable systematic, binding-mode-resolved evaluation, we curated AlloSet, a kinome-wide, time-split data set of kinase–ligand complexes annotated by the Modi and Dunbrack binding-mode nomenclature.^31^ Using this benchmark, we find that both diffusion docking (DiffDock-L) and cofolding methods exhibit a clear drop in performance for Type III/IV allosteric ligands relative to orthosteric ligands, consistent with the strong imbalance of available training data.^23,66,67^

We show that targeted fine-tuning of DiffDock-L on allosteric complexes can reshape the model sampling distribution and measurably improve allosteric pose recovery. In particular, fine-tuning yields statistically significant gains for Type III pose recovery (RMSD < 2 Å) and improves Type IV pocket localization (centroid <2 Å). Among the strategies explored, increased dropout helps preserve orthosteric performance, while translation/rotation-only fine-tuning (tr/rot_only) provides the strongest overall improvement for Type III/IV sampling. By contrast, temperature sampling and MD-based supersampling offered limited additional benefit in our setting.

Together, these results demonstrate that fine-tuning diffusion-based docking models is a practical and effective approach for improving performance in low-data allosteric regimes, and provide a concrete benchmark and workflow for adapting AI docking methods to underrepresented binding modes in kinase structure-based drug design.

In our work, we perform redocking to the original crystal structure. Future work can assess our docking model prospectively in a high-throughput screening setting.^72^ This adds another layer of complexity where it is important not only to dock and filter ligands appropriately but also to score and rank binders over nonbinders. We can gather the screening data in the literature for a retrospective screen and determine the hit-rate of our fine-tuned model.^11,13,73^ After using our fine-tuned model to dock a set of ligands, we can then use scoring function models that have been particularly tuned for the screening task to rank the binding poses.^65,74^ Ideally, the blind docking model will rely on learned features to classify the allosteric and orthosteric ligands, and a filtering step can then remove ligands more likely to be orthosteric. A model that is considered overfit to orthosteric binders for a particular protein class can be a beneficial attribute as a negative class filter. A proper test will contrast the performance of this blind docking approach with the performance of restricted-search space docking on both orthosteric and allosteric sites.

We also used DiffDock-L with the assumption that the receptor is rigid. Our previous work describes that only one particular conformation allowed allosteric docking success, primarily because it was a ligand bound at the allosteric site.^23^ AlphaFold2 structures can also be useful input receptors but may require methods to encourage conformational diversity, such as AlphaFold2-based methods that subsample the multiple sequence alignment^75–79^ and those that produce distributions of conformations^80–83^ or reveal cryptic pockets to receptor conformations primed for allosteric binding.^15,84,85^ A cofolding model tuned to produce diverse receptor conformations will circumvent the need to predict receptor structures ahead of time as well.

Other next steps can include evaluating the diversity^86–88^ and physical validity of the model predictions.^89,90^ Strategies like representation space probing can give qualitative interpretations of whether or not a deep learning model can be used for generalization tasks.^91^ Advancements can include creative architectures and training strategies. Concepts from the field of continual learning can be used to ensure that a deep learning model retains the capability to adapt without being retrained.^92^ Integrating first-principles or physics-based intuition into a machine learning model can guide performance in unexplored representation spaces.^93,94^ Lastly, a detailed collection of multimodal training data obtained through high-throughput noncompetitive screens or the utilization of pretrained models within translatable knowledge spaces can provide additional information for a model to learn from.^12,73,95,96^

## Supplementary Material



## Data Availability

All code is available at https://github.com/electrojustin/DiffDock-Fine-Tune. Data sets are available at doi: 10.5281/zenodo.17373043.

## Notes

Supporting Tables: distribution of the partial AlloSet broken down pocket labels; Supporting Figures: distribution of the full AlloSet broken down by ligand types and distance to noncanonical amino acids, test set results from tuning dropout, and the crystal ligand binding location embedding space; Supporting Methods: conformation-based loop modeling: to prepare receptors for the MD simulations, use a template-based approach, modeler, to model missing residue segments from similar protein conformations to the structure being remodeled (PDF)

## References

[1] Roskoski R. (2015). A historical
overview of protein kinases and their targeted small molecule inhibitors. Pharmcol. Res..

[2] Manning G., Whyte D. B., Martinez R., Hunter T., Sudarsanam S. (2002). The Protein
Kinase Complement of the Human Genome. Science.

[3] Noble M. E. M., Endicott J. A., Johnson L. N. (2004). Protein
Kinase Inhibitors: Insights
into Drug Design from Structure. Science.

[4] Lu X., Smaill J. B., Ding K. (2020). New Promise
and Opportunities for
Allosteric Kinase Inhibitors. Angew. Chem.,
Int. Ed..

[5] Pan Y., Mader M. M. (2022). Principles
of Kinase Allosteric Inhibition and Pocket
Validation. J. Med. Chem..

[6] Laufer S., Bajorath J. (2022). New Horizons in Drug
Discovery - Understanding and
Advancing Different Types of Kinase Inhibitors: Seven Years in Kinase
Inhibitor Research with Impressive Achievements and New Future Prospects. J. Med. Chem..

[7] Lu S., Shen Q., Zhang J. (2019). Allosteric Methods and Their Applications:
Facilitating the Discovery of Allosteric Drugs and the Investigation
of Allosteric Mechanisms. Acc. Chem. Res..

[8] Hardy J. A., Wells J. A. (2004). Searching for new
allosteric sites in enzymes. Curr. Opin. Struct.
Biol..

[9] Yamaguchi T., Yoshida T., Kurachi R. (2007). Identification of JTP-70902,
a p15INK4b-inductive compound, as a novel MEK1/2 inhibitor. Cancer Sci..

[10] Adrián F. J., Ding Q., Sim T., Velentza A., Sloan C., Liu Y., Zhang G., Hur W., Ding S., Manley P., Mestan J., Fabbro D., Gray N. S. (2006). Allosteric inhibitors
of Bcr-abl-dependent cell proliferation. Nat.
Chem. Biol..

[11] Rastelli G., Anighoro A., Chripkova M., Carrassa L., Broggini M. (2014). Structure-based
discovery of the first allosteric inhibitors of cyclin-dependent kinase
2. Cell Cycle.

[12] Faber E. B., Wang N., John K. (2023). Screening through Lead
Optimization of High Affinity, Allosteric Cyclin-Dependent Kinase
2 (CDK2) Inhibitors as Male Contraceptives That Reduce Sperm Counts
in Mice. J. Med. Chem..

[13] Faber E. B., Sun L., Tang J. (2023). Development of allosteric and selective CDK2
inhibitors for contraception with negative cooperativity to cyclin
binding. Nat. Commun..

[14] Vithani N., Zhang S., Thompson J. P., Patel L. A., Demidov A., Xia J., Balaeff A., Mentes A., Arnautova Y. A., Kohlmann A., Lawson J. D., Nicholls A., Skillman A. G., LeBard D. N. (2024). Exploration of Cryptic Pockets Using
Enhanced Sampling
Along Normal Modes: A Case Study of KRAS G12D. J. Chem. Inf. Model..

[15] Meller A., Ward M., Borowsky J., Kshirsagar M., Lotthammer J. M., Oviedo F., Ferres J. L., Bowman G. R. (2023). Predicting
locations of cryptic pockets from single protein structures using
the PocketMiner graph neural network. Nat. Commun..

[16] Laufkötter O., Hu H., Miljković F., Bajorath J. (2022). Structure- and Similarity-Based
Survey of Allosteric Kinase Inhibitors, Activators, and Closely Related
Compounds. J. Med. Chem..

[17] Nussinov R., Zhang M., Liu Y., Jang H. (2023). AlphaFold, allosteric,
and orthosteric drug discovery: Ways forward. Drug Discovery Today.

[18] Wenthur C. J., Gentry P. R., Mathews T. P., Lindsley C. W. (2014). Drugs for
Allosteric
Sites on Receptors. Annu. Rev. Pharmacol. Toxicol..

[19] Lu W., Wu Q., Zhang J., Rao J., Li C., Zheng S. (2022). TANKBind:
Trigonometry-Aware Neural NetworKs for Drug-Protein Binding Structure
Prediction. bioRxiv.

[20] Corso, G. ; Stärk, H. ; Jing, B. ; Barzilay, R. ; Jaakkola, T. DiffDock: Diffusion Steps, Twists, and Turns for Molecular Docking. 2022, arXiv:2210.01776. arXiv.org e-Print archive. https://arxiv.org/abs/2210.01776.

[21] Morehead, A. ; Giri, N. ; Liu, J. ; Neupane, P. ; Cheng, J. Deep Learning for Protein-Ligand Docking: Are We There Yet. 2025, arXiv:2405.14108. arXiv.org e-Print archive. https://arxiv.org/abs/2405.14108.

[22] Yang C., Chen E. A., Zhang Y. (2022). Protein-Ligand
Docking in the Machine-Learning
Era. Molecules.

[23] Chen E. A., Zhang Y. (2025). Can Deep Learning Blind Docking Methods
be Used to Predict Allosteric
Compounds?. J. Chem. Inf. Model..

[24] Durairaj J. (2024). PLINDER: The Protein-Ligand
Interactions Dataset and Evaluation Resource. bioRxiv.

[25] Škrinjar P., Eberhardt J., Durairaj J., Schwede T. (2025). Have Protein-Ligand
Co-folding Methods moved Beyond Memorisation?. bioRxiv.

[26] Corso, G. ; Deng, A. ; Fry, B. ; Polizzi, N. ; Barzilay, R. ; Jaakkola, T. Deep Confident Steps to New Pockets: Strategies for Docking Generalization. 2024, arXiv:2402.18396. arXiv.org e-Print archive. https://arxiv.org/abs/2402.18396.

[27] Kramer C., Chodera J., Damm-Ganamet K. L., Gilson M. K., Günther J., Lessel U., Lewis R. A., Mobley D., Nittinger E., Pecina A., Schapira M., Walters W. P. (2025). The Need for Continuing
Blinded Pose- and Activity Prediction Benchmarks. J. Chem. Inf. Model..

[28] Dodge, J. ; Ilharco, G. ; Schwartz, R. ; Farhadi, A. ; Hajishirzi, H. ; Smith, N. Fine-Tuning Pretrained Language Models: Weight Initializations, Data Orders, and Early Stopping. 2020, arXiv:2002.06305. arXiv.org e-Print archive. http://arxiv.org/abs/2002.06305.

[29] van
Linden O. P. J., Kooistra A. J., Leurs R., de Esch I. J. P., de Graaf C. (2014). KLIFS: A Knowledge-Based Structural Database To Navigate
Kinase-Ligand Interaction Space. J. Med. Chem..

[30] Kanev G. K., de Graaf C., Westerman B. A., de Esch I. J. P., Kooistra A. J. (2021). KLIFS:
an overhaul after the first 5 years of supporting kinase research. Nucleic Acids Res..

[31] Modi V., Dunbrack R. L. (2019). Defining a new nomenclature for the structures of active
and inactive kinases. Proc. Natl. Acad. Sci.
U.S.A..

[32] Modi V., Dunbrack R. L. (2022). Kincore: a web resource for structural
classification of protein kinases and their inhibitors. Nucleic Acids Res..

[33] Abramson J., Adler J., Dunger J. (2024). Accurate structure prediction
of biomolecular interactions with AlphaFold 3. Nature.

[34] Dana J. M., Gutmanas A., Tyagi N., Qi G., O’Donovan C., Martin M., Velankar S. (2019). SIFTS: updated
Structure Integration
with Function, Taxonomy and Sequences resource allows 40-fold increase
in coverage of structure-based annotations for proteins. Nucleic Acids Res..

[35] Xerxa E., Laufkötter O., Bajorath J. (2023). Systematic Analysis of Covalent and
Allosteric Protein Kinase Inhibitors. Molecules.

[36] Landrum, G. rdkit/rdkit: _03_1 (Q1 2020) Release, Zenodo, 2020. https://zenodo.org/record/3732262.

[37] Rogers D., Hahn M. (2010). Extended-Connectivity Fingerprints. J. Chem.
Inf. Model..

[38] Butina D.
U. (1999). Unsupervised
Data Base Clustering Based on Daylight’s Fingerprint and Tanimoto
Similarity: A Fast and Automated Way To Cluster Small and Large Data
Sets. J. Chem. Inf. Comput. Sci..

[39] Petrén H., Köllner T. G., Junker R. R. (2023). Quantifying chemodiversity considering
biochemical and structural properties of compounds with the R package
chemodiv. New Phytol..

[40] O’Boyle N. M., Banck M., James C. A., Morley C., Vandermeersch T., Hutchison G. R. (2011). Open Babel:
An open chemical toolbox. J. Cheminf..

[41] Song, Y. ; Sohl-Dickstein, J. ; Kingma, D. P. ; Kumar, A. ; Ermon, S. ; Poole, B. Score-Based Generative Modeling through Stochastic Differential Equations. 2021, arXiv:2011.13456. arXiv.org e-Print archive. https://arxiv.org/abs/2011.13456v2.

[42] Bortoli, V. D. ; Mathieu, E. ; Hutchinson, M. ; Thornton, J. ; Teh, Y. W. ; Doucet, A. Riemannian Score-Based Generative Modelling. 2022, arXiv:2202.02763. arXiv.org e-Print archive. http://arxiv.org/abs/2202.02763.

[43] Geiger, M. ; Smidt, T. e3nn: Euclidean Neural Networks. 2022, arXiv:2207.09453. arXiv.org e-Print archive. https://arxiv.org/abs/2207.09453.

[44] Lin Z., Akin H., Rao R., Hie B., Zhu Z., Lu W., Smetanin N., Verkuil R., Kabeli O., Shmueli Y., dos Santos Costa A., Fazel-Zarandi M., Sercu T., Candido S., Rives A. (2023). Evolutionary-scale
prediction of atomic-level protein structure with
a language model. Science.

[45] Meli R., Biggin P. C. (2020). spyrmsd: symmetry-corrected
RMSD calculations in Python. J. Cheminf..

[46] Wilson E. B. (1927). Probable
Inference, the Law of Succession, and Statistical Inference. J. Am. Stat. Assoc..

[47] Seabold, S. ; Perktold, J. In statsmodels: Econometric and Statistical Modeling with Python, 9th Python in Science Conference, 2010.

[48] Biewald, L. Experiment Tracking with Weights and Biases, 2020. https://www.wandb.com/.

[49] Liu Z., Li Y., Han L., Li J., Liu J., Zhao Z., Nie W., Liu Y., Wang R. (2015). PDB-wide collection
of binding data:
current status of the PDBbind database. Bioinformatics.

[50] Hinton, G. E. ; Srivastava, N. ; Krizhevsky, A. ; Sutskever, I. ; Salakhutdinov, R. R. Improving Neural Networks by Preventing Co-adaptation of Feature Detectors. 2012, arXiv:1207.0580. arXiv.org e-Print archive. http://arxiv.org/abs/1207.0580.

[51] Passaro S., Corso G., Wohlwend J., Reveiz M., Thaler S., Somnath V. R., Getz N., Portnoi T., Roy J., Stark H., Kwabi-Addo D., Beaini D., Jaakkola T., Barzilay R. (2025). Boltz-2: Towards Accurate
and Efficient Binding Affinity
Prediction. bioRxiv.

[52] Case, D. Amber 2023, 2023.

[53] Jurrus E., Engel D., Star K. (2018). Improvements to the
APBS biomolecular solvation software suite. Protein Sci..

[54] Wang J., Wolf R. M., Caldwell J. W., Kollman P. A., Case D. A. (2004). Development
and testing of a general amber force field. J. Comput. Chem..

[55] Wang J., Wang W., Kollman P. A., Case D. A. (2006). Automatic atom type
and bond type perception in molecular mechanical calculations. J. Mol. Graphics Modell..

[56] Maier J. A., Martinez C., Kasavajhala K., Wickstrom L., Hauser K. E., Simmerling C. (2015). ff14SB: Improving
the Accuracy of
Protein Side Chain and Backbone Parameters from ff99SB. J. Chem. Theory Comput..

[57] Mariani V., Biasini M., Barbato A., Schwede T. (2013). lDDT: a local
superposition-free
score for comparing protein structures and models using distance difference
tests. Bioinformatics.

[58] Biasini M., Schmidt T., Bienert S., Mariani V., Studer G., Haas J., Johner N., Schenk A. D., Philippsen A., Schwede T. (2013). OpenStructure: an integrated
software framework for
computational structural biology. Acta Crystallogr.,
Sect. D: Biol. Crystallogr..

[59] Cock P. J. A., Antao T., Chang J. T., Chapman B. A., Cox C. J., Dalke A., Friedberg I., Hamelryck T., Kauff F., Wilczynski B., de Hoon M. J. L. (2009). Biopython: freely
available Python tools for computational molecular biology and bioinformatics. Bioinformatics.

[60] Durbin, R. ; Eddy, S. R. ; Krogh, A. ; Mitchison, G. Biological Sequence Analysis: Probabilistic Models of Proteins and Nucleic Acids, 1st ed.; Cambridge University Press, 1998.

[61] Stein R. M., Yang Y., Balius T. E., O’Meara M. J., Lyu J., Young J., Tang K., Shoichet B. K., Irwin J. J. (2021). Property-Unmatched
Decoys in Docking Benchmarks. J. Chem. Inf.
Model..

[62] Su M., Yang Q., Du Y., Feng G., Liu Z., Li Y., Wang R. (2019). Comparative
Assessment of Scoring Functions: The CASF-2016
Update. J. Chem. Inf. Model..

[63] Ibrahim T. M., Bauer M. R., Boeckler F. M. (2015). Applying
DEKOIS 2.0 in structure-based
virtual screening to probe the impact of preparation procedures and
score normalization. J. Cheminf..

[64] Mysinger M. M., Carchia M., Irwin J. J., Shoichet B. K. (2012). Directory
of Useful
Decoys, Enhanced (DUD-E): Better Ligands and Decoys for Better Benchmarking. J. Med. Chem..

[65] Xia S., Gu Y., Zhang Y. (2025). Normalized Protein-Ligand Distance Likelihood Score
for End-to-End Blind Docking and Virtual Screening. J. Chem. Inf. Model..

[66] Nittinger E., Yoluk O., Tibo A., Olanders G., Tyrchan C. (2025). Co-folding,
the future of docking prediction of allosteric and orthosteric ligands. Artif. Intell. Life Sci..

[67] Olanders G., Testa G., Tibo A., Nittinger E., Tyrchan C. (2024). Challenge for Deep Learning: Protein Structure Prediction
of Ligand-Induced Conformational Changes at Allosteric and Orthosteric
Sites. J. Chem. Inf. Model..

[68] The OpenFold3 Team OpenFold3-preview, 2025. https://github.com/aqlaboratory/openfold-3.

[69] ApherisFold. https://www.apheris.com/apherisfold.

[70] Jain, A. N. ; Cleves, A. E. ; Walters, W. P. Deep-Learning Based Docking Methods: Fair Comparisons to Conventional Docking Workflows. 2024, arXiv:2412.02889. arXiv.org e-Print archive. http://arxiv.org/abs/2412.02889.

[71] Masters M. R., Mahmoud A. H., Lill M. A. D. (2024). Deep
Learning Models for Co-Folding
Learn the Physics of Protein-Ligand Interactions. bioRxiv.

[72] Lyu J., Kapolka N., Gumpper R. (2024). AlphaFold2 structures
guide prospective ligand discovery. Science.

[73] Martin M. P., Alam R., Betzi S., Ingles D. J., Zhu J.-Y., Schönbrunn E. (2012). A Novel Approach to the Discovery
of Small-Molecule
Ligands of CDK2. ChemBioChem.

[74] Shen C., Zhang X., Deng Y., Gao J., Wang D., Xu L., Pan P., Hou T., Kang Y. (2022). Boosting Protein-Ligand
Binding Pose Prediction and Virtual Screening Based on Residue-Atom
Distance Likelihood Potential and Graph Transformer. J. Med. Chem..

[75] Stein R. A., Mchaourab H. S. (2022). SPEACH_AF: Sampling protein ensembles
and conformational
heterogeneity with Alphafold2. PLoS Comput.
Biol..

[76] del
Alamo D., Sala D., Mchaourab H. S., Meiler J. (2022). Sampling alternative conformational states of transporters
and receptors with AlphaFold2. eLife.

[77] Monteiro
da Silva G., Cui J. Y., Dalgarno D. C., Lisi G. P., Rubenstein B. M. (2024). High-throughput prediction of protein conformational
distributions with subsampled AlphaFold2. Nat.
Commun..

[78] Sala D., Hildebrand P. W., Meiler J. (2023). Biasing AlphaFold2 to predict GPCRs
and kinases with user-defined functional or structural properties. Front. Mol. Biosci..

[79] Al-Masri C., Trozzi F., Lin S.-H., Tran O., Sahni N., Patek M., Cichonska A., Ravikumar B., Rahman R. (2023). Investigating the conformational
landscape of AlphaFold2-predicted
protein kinase structures. Bioinf. Adv..

[80] Zheng S., He J., Liu C. (2024). Predicting
equilibrium distributions for molecular
systems with deep learning. Nat. Mach. Intell..

[81] Jing, B. ; Berger, B. ; Jaakkola, T. AlphaFold Meets Flow Matching for Generating Protein Ensembles. 2024, arXiv:2402.04845. arXiv.org e-Print archive. http://arxiv.org/abs/2402.04845.

[82] Pang Y. T., Kuo K. M., Yang L., Gumbart J. C. (2025). DeepPath: Overcoming
data scarcity for protein transition pathway prediction using physics-based
deep learning. bioRxiv.

[83] Bhakat S., Vats S., Mardt A., Degterev A. (2025). Generalizable Protein
Dynamics in Kinases: Physics is the key. bioRxiv.

[84] Vats S., Bobrovs R., Söderhjelm P., Bhakat S. (2024). AlphaFold-SFA: Accelerated
sampling of cryptic pocket opening, protein-ligand binding and allostery
by AlphaFold, slow feature analysis and metadynamics. PLoS One.

[85] Meller A., Bhakat S., Solieva S., Bowman G. R. (2023). Accelerating
Cryptic
Pocket Discovery Using AlphaFold. J. Chem. Theory
Comput..

[86] Sehwag, V. ; Hazirbas, C. ; Gordo, A. ; Ozgenel, F. ; Ferrer, C. C. Generating High Fidelity Data from Low-density Regions using Diffusion Models. 2022, arXiv:2203.17260. arXiv.org e-Print archive. http://arxiv.org/abs/2203.17260.

[87] Corso, G. ; Xu, Y. ; de Bortoli, V. ; Barzilay, R. ; Jaakkola, T. Particle Guidance: non-I.I.D. Diverse Sampling with Diffusion Models. 2023, arXiv:2310.13102. arXiv.org e-Print archive. http://arxiv.org/abs/2310.131020.

[88] Tossou, P. ; Wognum, C. ; Craig, M. ; Mary, H. ; Noutahi, E. Real-World Molecular Out-Of-Distribution: Specification and Investigation ChemRxiv, 2023. 10.1021/acs.jcim.3c01774 PMC10865358 38300258

[89] Buttenschoen, M. ; Morris, G. M. ; Deane, C. M. PoseBusters: AI-based docking methods fail to generate physically valid poses or generalise to novel sequences. 2023, arXiv:2308.05777. arXiv.org e-Print archive. http://arxiv.org/abs/2308.05777. 10.1039/d3sc04185a PMC10901501 38425520

[90] Masters M. R., Mahmoud A. H., Lill M. A. (2025). Investigating
whether deep learning
models for co-folding learn the physics of protein-ligand interactions. Nat. Commun..

[91] Wan Y., Wu J., Hou T., Hsieh C.-Y., Jia X. (2025). Multi-channel learning
for integrating structural hierarchies into context-dependent molecular
representation. Nat. Commun..

[92] Dohare S., Hernandez-Garcia J. F., Lan Q., Rahman P., Mahmood A. R., Sutton R. S. (2024). Loss of plasticity in deep continual
learning. Nature.

[93] Wang Y., Fass J., Kaminow B., Herr J. E., Rufa D., Zhang I., Pulido I., Henry M., Chodera J. D. (2022). End-to-End Differentiable
Molecular Mechanics Force Field Construction. Chem. Sci..

[94] Rufa, D. A. ; Macdonald, H. E. B. ; Fass, J. ; Wieder, M. ; Grinaway, P. B. ; Roitberg, A. E. ; Isayev, O. ; Chodera, J. D. Towards chemical accuracy for alchemical free energy calculations with hybrid physics-based machine learning/molecular mechanics potentials bioRxiv, 2020 10.1101/2020.07.29.227959v1.

[95] Fang Y., Zhang Q., Zhang N., Chen Z., Zhuang X., Shao X., Fan X., Chen H. (2023). Knowledge graph-enhanced
molecular contrastive learning with functional prompt. Nat. Mach. Intell..

[96] Hayes T., Rao R., Akin H. (2025). Simulating
500 million years of evolution with
a language model. Science.

