# Supplementary material for: Fine-Tuning DiffDock‑L for Allosteric Kinase Docking
Source: J Chem Inf Model. 2026 Mar 4;66(6):3036–48. doi: 10.1021/acs.jcim.5c02846 (PMC13014456; doi:10.1021/acs.jcim.5c02846)
Supplement: Supplementary file 1 [file ci5c02846_si_001.pdf]

Supporting Information: Fine-Tuning DiffDock-L for Allosteric Kinase Docking

Eric Chen,[†,¶] Justin Green,[†,§] and Yingkai Zhang[*,‡,‖]

†*These authors contributed equally to this work.*

‡*Simons Center for Computational Physical Chemistry at New York University, New York, NY 10003, United States*

¶*Department of Chemistry, New York University, New York, NY 10003, United States*

§*Department of Biology, New York University, New York, NY 10003, United States*

E-mail: yingkai.zhang@nyu.edu

Supporting Tables and Figures

Table S1: Breakdown of the AlloSet by the pocket definitions of Laufkötter dataset[1,2]

Pocket	Train/Val	Test
B	72	16
C	32	5
D	25	2
E	2	3
F	4	2
G	3	0
H	14	8
H+A	18	0
J	7	2
L	2	0

Note: Because AlloSet is a superset of the Laufötter dataset, not all AlloSet datapoints are reflected here.

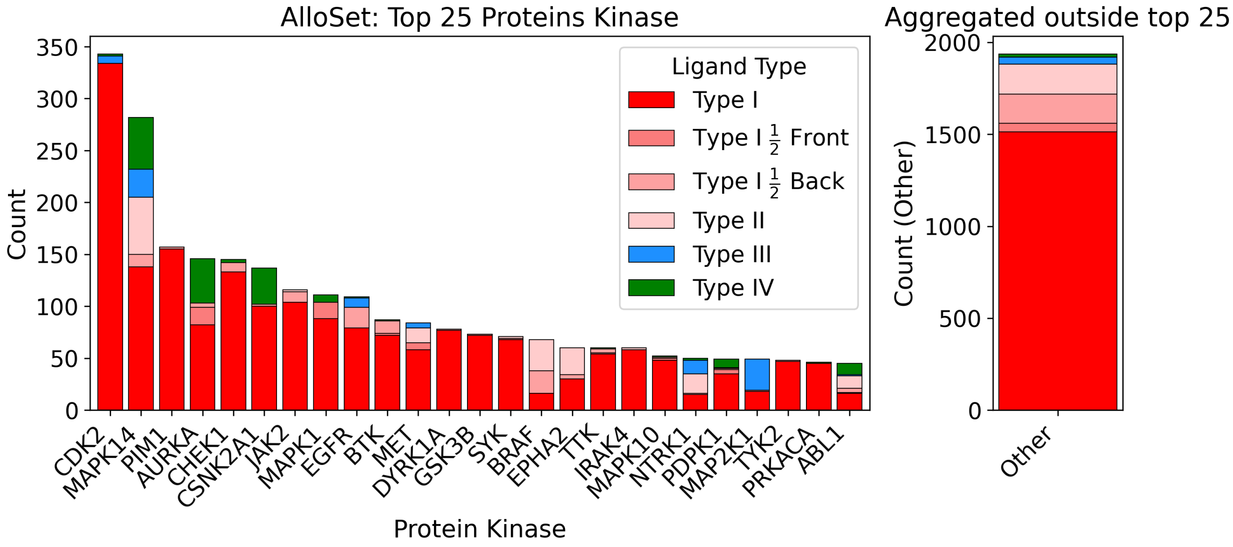

Figure S1: Distribution of the top 25 AlloSet kinases broken down by the Modi and Dunbrack ligand types and de-duplicated by pairs of UniProt ID and ligand name. The kinases outside of the top 25 are aggregated in a separate column

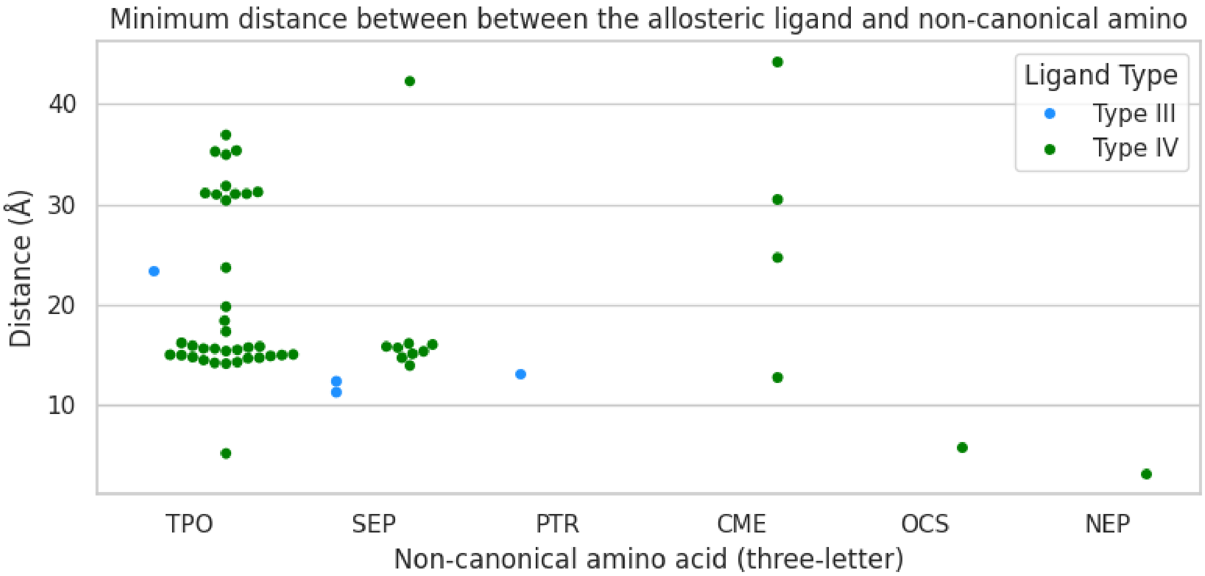

Figure S2: Distribution of the minimum distance between non-canonical amino acids and the bound ligand broken down by ligand type. The only non-canonical amino acid–ligand distance pair below 5 Å is NEP–B8Z in PDB: 7xc1 chain A.

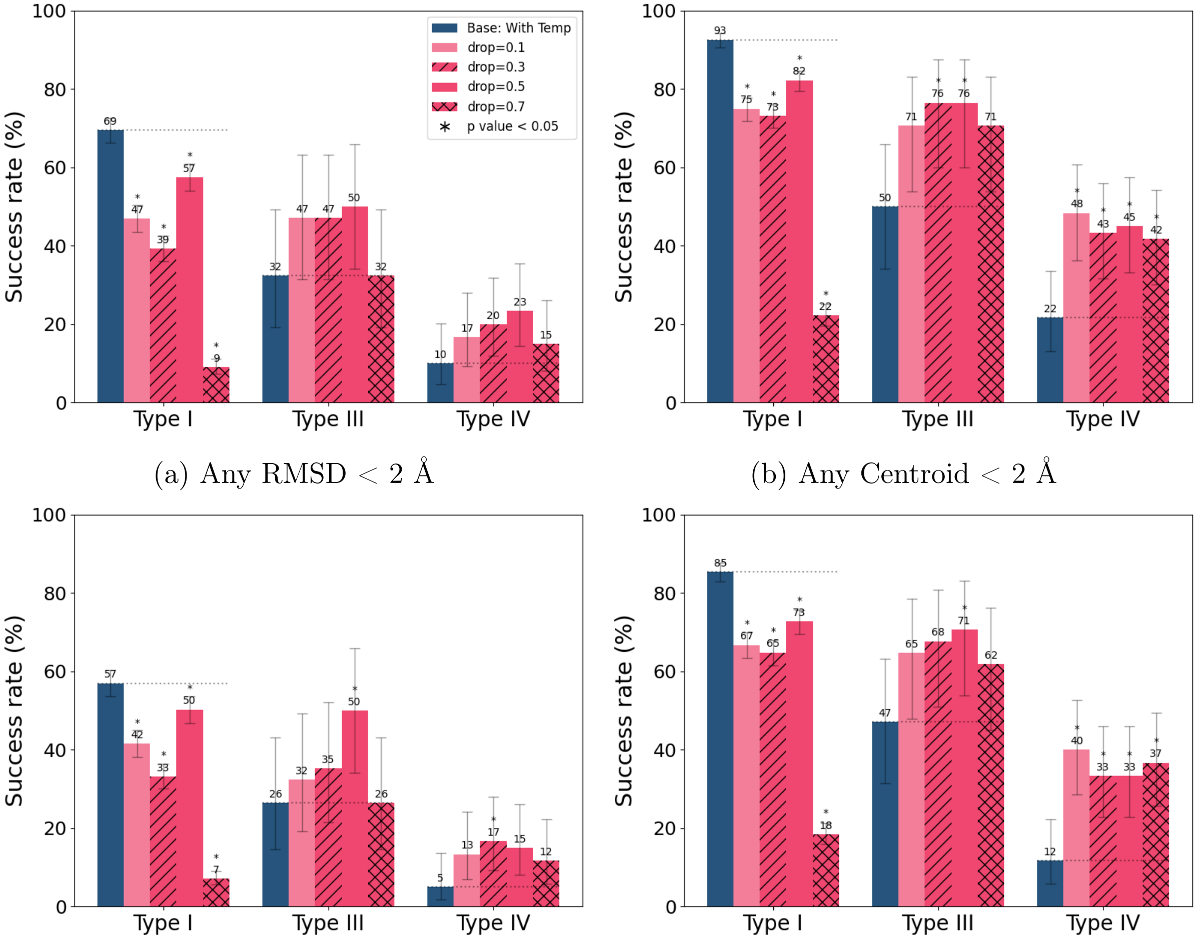

(a) Any RMSD < 2 Å (b) Any Centroid < 2 Å

(c) Top 1 RMSD < 2 Å (d) Top 1 Centroid < 2 Å

Figure S3: Plots showing the fraction of fine-tuned models with varying dropouts predicted complexes in the test set that satisfy the requirements (a) if any of the 10 sampled poses that result in RMSD < 2 Å or (b) centroid distance < 2 Å and (c) if the top 1 confidence poses result in RMSD < 2 Å or (d) centroid distance < 2 Å. The blue bars indicate that the baseline DiffDock-L models with and without temperature sampling. The unhashed plots reflect the performance reported in the main text and the hashed plots indicate additional experiments tuning dropout. The dotted line indicates the performance of DiffDock-L baseline without temperature sampling, error bars denote 95% confidence intervals, an asterisk (*) denotes statistical significance ($p < 0.05$) compared with the baseline condition without temperature sampling.

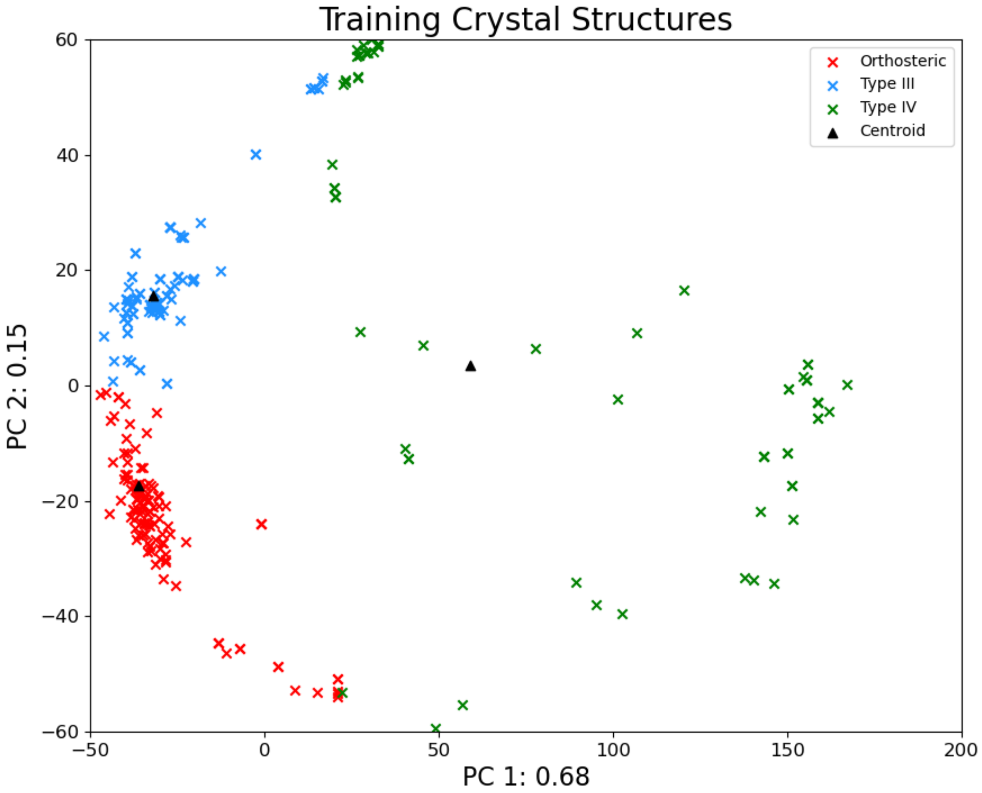

Figure S4: PCA of kinase-ligand fingerprints for crystal structures. The X- and Y-axis reflect the first and second principal components, respectively, and is labeled with explained variance ratio. The color of the marker indicates the Modi and Dunbrack labeled binding modality. Red indicates an orthosteric inhibitor, while blue and green indicate Type III and Type IV inhibitors respectively. Black triangles are the centroids of each binding modality.

Supporting Methods

Conformation-based Loop Modeling

To prep the ligand bound crystal structures for molecular dynamics (MD) simulations, we perform *conformation-based loop modeling*. This selects similar protein conformations (sample structures) to the structure to be remodelled (reference structure) and uses a template-based approach, Modeller, to model missing residues segments.[3,4] When selecting template structures, we aimed to use structures of kinases that were in conformations as near as possible to the structure being remodelled. For this purpose, we used the Mininum Distance Matrix Representation (MDMR) to robustly cluster and classify protein structures by their respective conformations.[5] This method is useful for repairing structures that have similar conformations from the same or related protein.

We first use the MDMR of the KLIFS residues to embed the receptor structures of all of the kinase structures in the AlloSet.[6,7] We impute the missing distances in the matrix using the k-Nearest Neighbors imputer in scikit-learn.[8,9] These matrices are then input into the dimension reduction method Uniform Manifold Approximation and Projection (UMAP)[10] with `n_neighbors=25`, `min_dist=0.3` and `metric='correlation'` and then clustered using the Hierarchical Density-Based Spatial Clustering of Applications with Noise (HDBSCAN) algorithm with `min_cluster_size=10` and `cluster_selection_epsilon=1`.[11,12] This results in 33 clusters (**Supplemental Figure S5**).

We determine the missing residues for all of structures in the AlloSet training set and group adjacent residues into segment gaps. Any unresolved terminal residues or intrinsically-disordred regions are excluded.[13] Any non-canonical amino acid is converted back to the original amino acid as defined by the UniProt sequence.

To assign sample structures to reference structures, we iterate over the closest UMAP-projected datapoints by Euclidean distance. For each sample, we check whether the sample structure has resolved the corresponding gaps in the reference structure by using the KLIFS

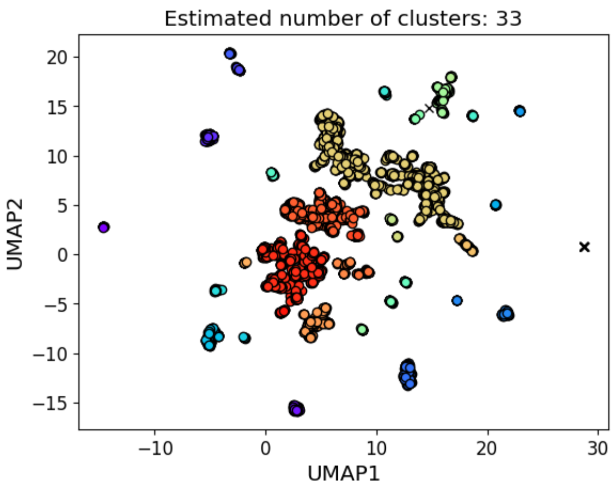

Figure S5: UMAP and HDBSCAN clusters of the AlloSet training set structures. Each structure is represented by their Mininum Distance Matrix Representation (MDMR) of the KLIFS residues and is colored by their respective HDBSCAN clustering labels. Noisy and unclustered data points are represented with an ×.

residues and the alignment points between structures. We then align the sample sequence to the reference sequence with the KLIFS residues as anchors using COBALT.[14] Because the KLIFS residues provide a structural alignment of the catalytic region, we anticipate that the corresponding regions in between KLIFS residues are likely to provide similar structural roles. We iterate through the samples until each gap in the reference structure is accounted for.

The reconstruction of the missing loop regions uses an adapted version of the `AutoModel` class in Modeller. A model with the corrected sequence is first quickly rebuilt. The missing regions are reconstructed based on the assigned template structures. 5 models are generated with hydrogens and then optimized with the built-in protocols using variable target function method[15] and molecular dynamics (MD) refinement with simulated annealing.[4,16] The best model by lowest DOPE score is selected for MD.

References

(1) Laufkötter, O.; Hu, H.; Miljković, F.; Bajorath, J. Structure- and Similarity-Based Survey of Allosteric Kinase Inhibitors, Activators, and Closely Related Compounds. *J. of Med. Chem.* **2022**, *65*, 922–934.

(2) Xerxa, E.; Laufkötter, O.; Bajorath, J. Systematic Analysis of Covalent and Allosteric Protein Kinase Inhibitors. *Mol.* **2023**, *28*, 5805.

(3) Šali, A.; Blundell, T. L. Comparative Protein Modelling by Satisfaction of Spatial Restraints. *J. of Mol. Biology* **1993**, *234*, 779–815.

(4) Webb, B.; Sali, A. Comparative Protein Structure Modeling Using MODELLER. *Curr. Protoc. in Bioinform.* **2016**, *54*, 5.6.1–5.6.37.

(5) Chen, E. A.; Zhang, Y. Can Deep Learning Blind Docking Methods be Used to Predict Allosteric Compounds? *J. of Chem. Inf. Model.* **2025**, *65*, 3737–3748.

(6) van Linden, O. P. J.; Kooistra, A. J.; Leurs, R.; de Esch, I. J. P.; de Graaf, C. KLIFS: A Knowledge-Based Structural Database To Navigate Kinase–Ligand Interaction Space. *J. of Med. Chem.* **2014**, *57*, 249–277.

(7) Kanev, G. K.; de Graaf, C.; Westerman, B. A.; de Esch, I. J. P.; Kooistra, A. J. KLIFS: an overhaul after the first 5 years of supporting kinase research. *Nucl. Acids Res.* **2020**, *49*, D562–D569.

(8) Pedregosa, F. et al. Scikit-learn: Machine Learning in Python. 2018; `http://arxiv.org/abs/1201.0490`.

(9) Troyanskaya, O.; Cantor, M.; Sherlock, G.; Brown, P.; Hastie, T.; Tibshirani, R.; Botstein, D.; Altman, R. B. Missing value estimation methods for DNA microarrays. *Bioinform.* **2001**, *17*, 520–525.

(10) McInnes, L.; Healy, J.; Melville, J. UMAP: Uniform Manifold Approximation and Projection for Dimension Reduction. 2020; `http://arxiv.org/abs/1802.03426`.

(11) Campello, R. J. G. B.; Moulavi, D.; Sander, J. In *Advances in Knowledge Discovery and Data Mining*; Hutchison, D. et al. , Eds.; Springer Berlin Heidelberg: Berlin, Heidelberg, 2013; Vol. 7819; pp 160–172.

(12) Malzer, C.; Baum, M. A Hybrid Approach To Hierarchical Density-based Cluster Selection. 2020 IEEE International Conference on Multisensor Fusion and Integration for Intelligent Systems (MFI). 2020; pp 223–228.

(13) Mészáros, B.; Erdős, G.; Dosztányi, Z. IUPred2A: context-dependent prediction of protein disorder as a function of redox state and protein binding. *Nucl. Acids Res.* **2018**, *46*, W329–W337.

(14) Papadopoulos, J. S.; Agarwala, R. COBALT: constraint-based alignment tool for multiple protein sequences. *Bioinform.* **2007**, *23*, 1073–1079.

(15) Braun, W.; Gō, N. Calculation of protein conformations by proton-proton distance constraints: A new efficient algorithm. *J. of Mol. Biology* **1985**, *186*, 611–626.

(16) Clore, G. M.; Brünger, A. T.; Karplus, M.; Gronenborn, A. M. Application of molecular dynamics with interproton distance restraints to three-dimensional protein structure determination: A model study of crambin. *J. of Mol. Biology* **1986**, *191*, 523–551.
